# Optoelectronic Properties of Tin–Lead Halide Perovskites

**DOI:** 10.1021/acsenergylett.1c00776

**Published:** 2021-06-10

**Authors:** Kimberley J. Savill, Aleksander M. Ulatowski, Laura M. Herz

**Affiliations:** † Clarendon Laboratory, Department of Physics, University of Oxford, Parks Road, Oxford OX1 3PU, U.K.; ‡ TUM Institute for Advanced Study, 85748 Garching bei München, Germany

## Abstract

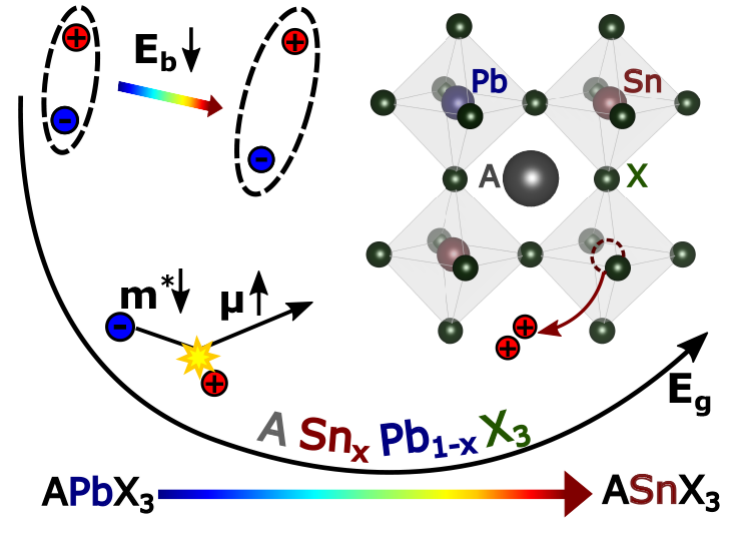

Mixed tin–lead halide perovskites have recently emerged as highly promising materials for efficient single- and multi-junction photovoltaic devices. This Focus Review discusses the optoelectronic properties that underpin this performance, clearly differentiating between intrinsic and defect-mediated mechanisms. We show that from a fundamental perspective, increasing tin fraction may cause increases in attainable charge-carrier mobilities, decreases in exciton binding energies, and potentially a slowing of charge-carrier cooling, all beneficial for photovoltaic applications. We discuss the mechanisms leading to significant bandgap bowing along the tin–lead series, which enables attractive near-infrared bandgaps at intermediate tin content. However, tin-rich stoichiometries still suffer from tin oxidation and vacancy formation which often obscures the fundamentally achievable performance, causing high background hole densities, accelerating charge-carrier recombination, lowering charge-carrier mobilities, and blue-shifting absorption onsets through the Burstein–Moss effect. We evaluate impacts on photovoltaic device performance, and conclude with an outlook on remaining challenges and promising future directions in this area.

Metal halide perovskites have recently emerged as an exciting new class of semiconductors for solar energy generation, with device efficiencies now competing with those of commercial silicon cells.^1^ To date, highest reported power conversion efficiencies (PCEs) for single-junction devices have relied on the exceptional performance of lead-based perovskites, which offer strong absorption,^2^ long charge-carrier lifetimes and diffusion lengths,^3−5^ and high defect tolerance.^6−8^ However, the lowest bandgaps attainable for lead halide perovskites are around 1.5 eV,^9^ higher than the value of ∼1.3 eV required for maximum theoretical efficiencies of single-junction devices.^10,11^ Together with concerns about toxicity of lead in its soluble form,^12^ these issues have led to increased research on alternative metal halide semiconductors.^13−16^

Currently, the most promising materials to address these issues are mixed-metal tin–lead halide perovskites of stoichiometry ASn_*x*_Pb_1–*x*_X_3_, where the A-site is typically occupied by formamidinium (FA^+^), methylammonium (MA^+^), cesium (Cs^+^), or a mixture thereof, and the X-site mostly by iodide (to achieve lowest bandgaps), but bromide inclusion has also been reported.^17^ Iodide-rich versions of these materials offer bandgap tunability between 1.2 and 1.6 eV (see below) and have become the leading choice for the narrow-bandgap absorber layer in all-perovskite tandem cells, as summarized in recent reviews.^17−19^ Such devices combine the enhanced efficiency of a multi-junction architecture with lower processing temperatures and greater compositional tunability compared with perovskite-silicon tandem cells. The highest reported power conversion efficiencies of all-perovskite tandem cells incorporating mixed tin–lead halide perovskites have now reached 25%^20^ for 4-terminal cells, and 25.6% for 2-terminal cells,^21^ while single-junction cells with PCEs near 21% have also just been reported.^21,22^

While tin–lead halide perovskites have clearly excelled in photovoltaic devices, knowledge of their underlying optoelectronic properties is still emerging. Here, one obstacle has been the interplay of intrinsic (fundamental) effects and extrinsic effects deriving from their defect chemistry. Such issues are particularly prominent in the tin-rich stoichiometries, in which tin in its 2+ state is unstable to oxidation and vacancy formation,^23−25^ resulting in a high background doping density of holes.^26−29^ As a result, the field is still struggling to probe the truly fundamental limits of optoelectronic performance, which may be masked by such unintentional doping. This review aims to unravel such effects, providing a clear view of how fundamental and extrinsic mechanisms shape the optoelectronic properties of tin–lead halide perovskites. We explain the underlying scientific concepts governing the peculiar effect of bandgap bowing in these materials, and discuss how exciton binding energies, charge-carrier cooling, and maximum attainable charge-carrier mobilities vary along the tin–lead series. We further summarize how the defect chemistry of tin–lead halide perovskites often dominates their optoelectronic properties, accelerating charge-carrier recombination, lowering charge-carrier mobilities, and blue-shifting absorption onsets through the Burstein–Moss effect. For each of these phenomena, we discuss the impact on photovoltaic device performance, and conclude with an outlook on the remaining challenges and promising future areas of research for tin–lead halide perovskites. We hope the analysis provided will allow for a complete understanding of these materials that facilitates their implementation in photovoltaic and light-emitting devices.

## 1 Oxidation of Sn^2+^, Tin Vacancy Formation, and Self-Doping

One profound difference between tin halide perovskites and their lead halide counterparts derives from the instability of Sn^2+^ against oxidation to Sn^4+^ and the propensity for tin vacancy formation. This unfavorable defect chemistry has a significant extrinsic influence on the optoelectronic properties of mixed tin–lead perovskites, in particular their tin-rich compositions, which will be examined in detail in this review. To aid discussion, we therefore first briefly review the mechanisms governing tin oxidation, as well as tin vacancy formation and its unintentional consequence of introducing a large density of background holes.

From a basic chemical perspective, lead is considered to be most stable in the 2+ oxidation state; however, such stability decreases for lighter group 14 metals, with the result that tin favors the 4+ oxidation state and can be readily oxidized in tin halide perovskites^30,31^ as well as in tin precursor solutions.^32,33^ Such chemical instability of tin-rich metal halide perovskites introduces significant decomposition pathways, which also render them less stable to oxygen and moisture than lead halide perovskites.^24,25^ The oxidation of Sn^2+^ may form part of a chemical conversion that introduces secondary phases within an ASnI_3_ perovskite, such as SnI_4_ or the vacancy-ordered compound A_2_SnI_6_, in which tin exists in its Sn^4+^ form.^24,34^ Introduction of oxygen favors such processes, resulting in facile decomposition pathways with end products of AI, SnO_2_, and SnI_4_,^24^ making these materials profoundly unstable in air.^31^ Substitution of lead for tin will gradually raise hurdles to metal oxidation, such that majority-lead compositions instead revert to their precursor components as part of their favored decomposition pathway.^24,35^

**Figure 1:**
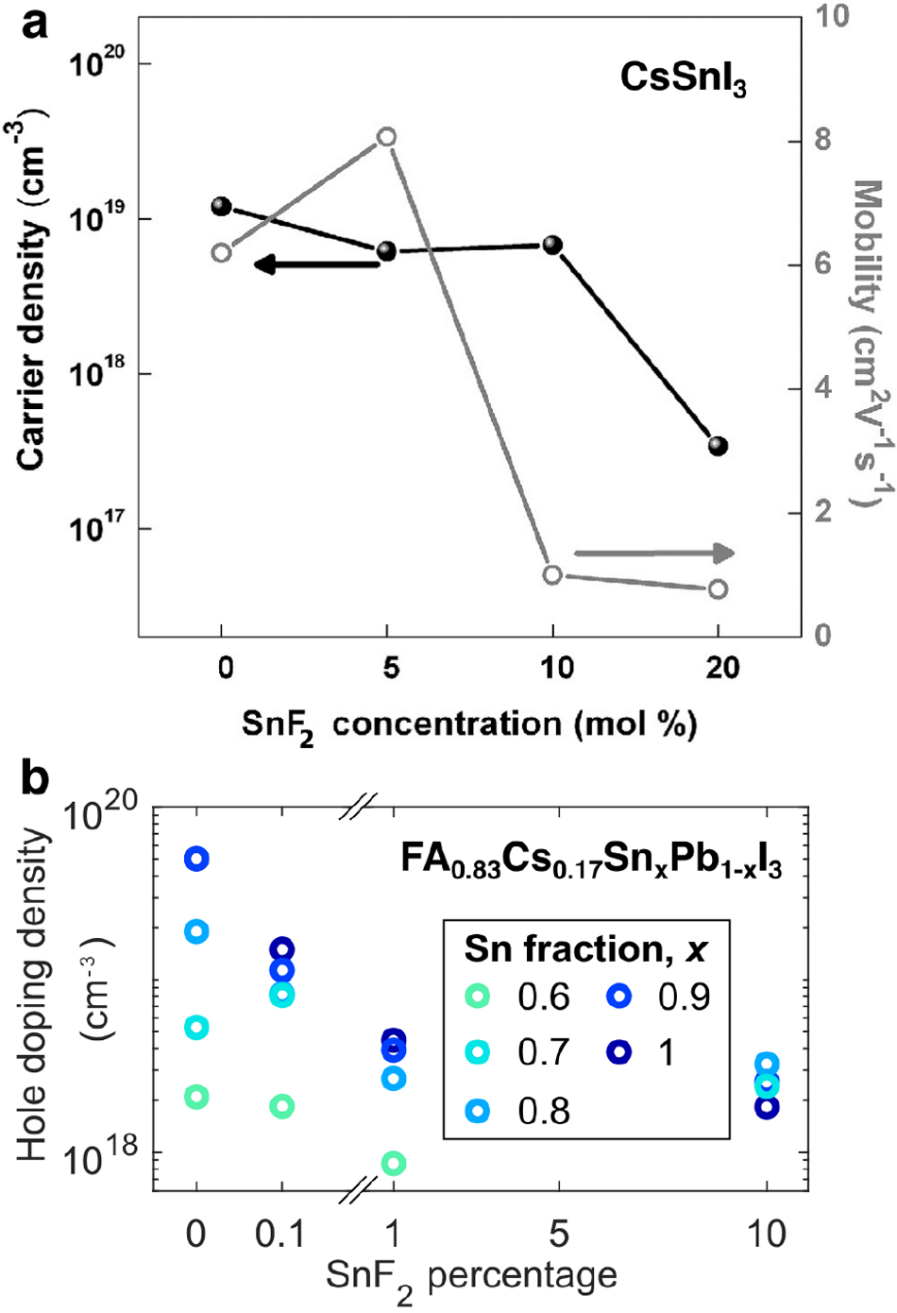
Background hole doping density in tin–lead iodide perovskite films as a function of the relative percentages of SnF_2_ included in the precursor solutions with respect to SnI_2_: (a) For CsSnI_3_, extracted from Hall effect measurements which also yield values shown for the hole mobility. [Reprinted with permission from ref (28). Copyright 2014 John Wiley and Sons.] (b) For FA_0.83_Cs_0.17_Sn_*x*_Pb_1–*x*_I_3_ with high tin content *x* ranging between 60 and 100%, determined from an analysis of THz dark conductivity spectra. [Adapted with permission from ref (42). Copyright 2020 John Wiley and Sons.] We note that slight variations in trends with SnF_2_ percentage are visible between studies and stoichiometries, which are most likely related to measurement uncertainties, sample-to-sample variations, and different extents of sample exposure to ambient environment.

From an electronic perspective, the underperformance of tin halide perovskites is more readily understood within the concept of tin vacancy formation. Tin iodide perovskites exhibit much lower ionization energies than their lead-based counterparts,^35^ because of their reduced spin–orbit coupling (tin is lighter than lead).^36,37^ Density functional theory calculations have shown that tin vacancies are highly stable under these conditions,^23,35,38^ and may also create a locally iodine-rich environment that promotes the oxidation of Sn^2+^ and the chemical conversions described above.^35^ Easily formed, both tin vacancies and iodide interstitials generate defect levels just below the valence band edge, where they capture valence band electrons, effectively releasing free holes. Such unintentional p-type doping is therefore ubiquitous in tin iodide perovskites, for which our literature survey^26−29,31,39−42^ finds experimentally reported hole densities to range predominantly around 10^17^–10^20^ cm^–3^ (see Figure 1 for some examples). When metal content is reduced to 70–90% tin by substitution with lead, reported values^42^ fall to around 10^17^–10^18^ cm^–3^, and to 10^15^–10^17^ cm^–3^ for 50–60% tin content.^5,20,21,32,42−46^ Once lead is the predominant metal, background doping densities are rarely mentioned for tin–lead perovskites, meaning they most likely fall well below the density of photoexcited charge-carriers under solar illumination conditions (10^15^–10^16^ cm^–3^)^47^ and therefore have relatively little impact on photovoltaic device operation. Clearly, lead-rich tin–lead halide perovskites exhibit an inherently lower propensity for metal vacancy formation and hole doping, which has been ascribed to increased hurdles to oxidation^24,30^ following a drop in the valence band maximum (a rise in ionization energy).^35^

As discussed in detail below, the presence of tin vacancies and the resulting unintentional hole doping detrimentally affects optoelectronic performance of tin–lead halide perovskites. A wide range of strategies has therefore been explored to prevent such effects, including the use of additives to act as reducing agents or tin sources, control of crystallization, partial ion substitution, and reduced dimensionality. A full discussion of these is beyond the scope of this review, and we refer the reader to several existing reviews for full details.^25,48−50^ Currently, the most frequently utilized approach involves the addition of SnF_2_ to the precursor solution, which reduces the formation prospects of tin-poor stoichiometries.^28,29,42^ As Figure 1 illustrates, SnF_2_ addition to tin–lead halide perovskites considerably lowers the density of background holes (p-type doping),^28,29,42^ in particular for tin-rich stoichiometries. We note that as such mitigation strategies against tin oxidation and vacancy formation are further refined, the intrinsic optoelectronic properties discussed in this review are expected to become more prominent and relevant to device performance.

## 2 Bandgap Tunability and Bowing

**Figure 2:**
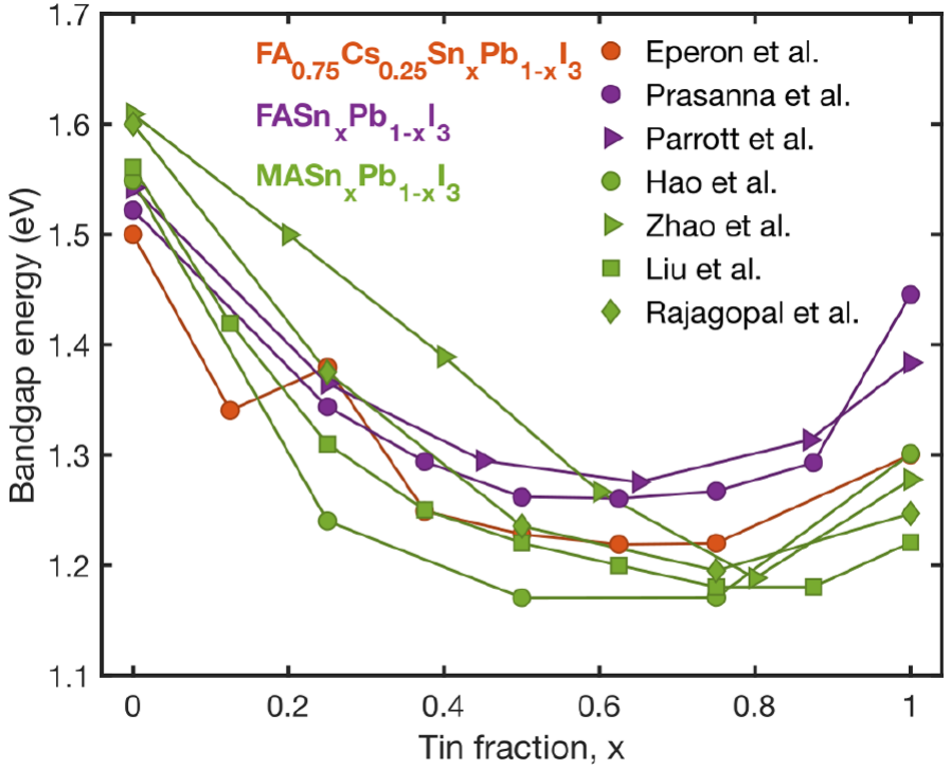
Values of the bandgap energy at room temperature extracted from a range of literature studies^51−57^ for tin–lead iodide perovskites ASn_*x*_Pb_1–*x*_I_3_, where A is methylammonium (MA), formamidinium (FA), or cesium (Cs), or a mixture thereof, as indicated in the legend.

One prominent reason for tin–lead iodide perovskites being particularly attractive for photovoltaic applications is their bandgap tunability in the range of 1.2–1.6 eV, which encompasses values required for optimum single-cell efficiencies (∼1.3 eV)^10,11^ as well as suitable low-bandgap candidates for bottom cells in all-perovskite tandem devices.^17,18^ As Figure 2 illustrates, the wide range of bandgap energies offered by the ASn_*x*_Pb_1–*x*_I_3_ series partly results from significant bandgap bowing, meaning that the alloyed perovskites exhibit lower bandgaps than either of the “parent” compositions APbI_3_ or ASnI_3_.^51−57^ In addition, smaller variations in bandgap can be obtained from substitution of A-cations (see Figure 2) or halide anions. Mixed tin–lead iodide perovskites thus offer a highly attractive bandgap tuning range for multi-junction photovoltaic devices, light-emitting diodes, and photodetectors.

Bandgap bowing is not uncommon in alloyed semiconductors, including, e.g., in GaN_*x*_As_1–*x*_, GaAs_*x*_Sb_1–*x*_, CdSe_*x*_Te_1–*x*_, and ZnS_*x*_Te_1–*x*_.^58^ To quantify the extent of such bowing, the bandgap bowing parameter *b* is usually defined by the following equation:
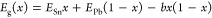
1where *E*_g_(*x*) is the bandgap energy of the mixed tin–lead halide perovskites, *E*_Sn_ and *E*_Pb_ are the bandgaps of the tin-only and lead-only halide perovskites, respectively, and *x* is the fraction of tin included on the metal site. The first two terms represent a linear change in bandgap with composition between the two end points, while the third term captures any quadratic (parabolic) deviation from such linearity. While bandgap bowing is prominent in tin–lead halide perovskites, yielding experimental values of *b* for a range of A cation compositions of between 0.5 and 0.9 at room temperature,^42,53,57,59^ it is almost absent for metal halide perovskites upon halide substitution.^9,60^ Understanding the origins of bandgap bowing in tin–lead perovskites is therefore helpful to the realization of lowest achievable bandgaps. The emerging literature consensus points toward bandgap bowing in mixed tin–lead perovskites arising mostly from a combination of structural relaxation effects and chemical effects mediated by spin–orbit coupling.^53,57,61−64^ Structural relaxation accommodates the random placement of differently sized lead and tin ions throughout the lattice by bond bending, as a result of which the structure varies locally and is not well described by an average value.^53^ Because changes in the metal halide bond angle in these perovskites alter the bandgap, this structural relaxation contributes significantly to bandgap bowing, as has been identified both from first-principles calculations^61^ and through experiments.^53,57,62^ The magnitude of bowing varies somewhat with the choice of A cation and halide anion, which mediate structural relaxation effects through microstrain in the crystal structure.^57^ Chemical effects, wherein the atomic orbitals of tin and lead that respectively form the valence band and conduction band edges are mismatched in energy, also have a significant influence on bandgap bowing.^57,63^ The contribution of spin–orbit coupling to bandgap bowing,^61^ previously disputed,^63^ can also be understood in relation to chemical effects as spin–orbit coupling enhances the influence of lead on the conduction band minimum so that the mismatch in energy between lead and tin orbitals becomes significant.^64^

In addition to the local variations in structure which contribute to bandgap bowing, the metal ratio in mixed tin–lead perovskites affects overall crystal structure. The smaller size of tin cations compared to lead cations results in decreasing lattice parameter values as tin content increases, as has been observed via shifts in X-ray diffraction peak position with tin content.^42,52,55,56,65,66^ In cases where the lead-only and tin-only perovskite compositions have different crystal structures, a transition between structures must occur at intermediate tin content. In MASn_1–*x*_Pb_*x*_I_3_ perovskites, a change from the tetragonal structure encountered for lead-rich compositions to a (pseudo)cubic structure at the tin-rich end has been observed to take place at 50% tin content.^30,54−56^ By contrast, increasing tin content in perovskites with an otherwise pseudocubic structure can lead to increasing tetragonal distortion as a result of tin vacancy formation and associated lattice strain, an extrinsic effect which is suppressed by additives to control oxidation of tin.^42^ The trends in crystal structure across the tin–lead compositional range are therefore influenced by the degree to which unwanted tin vacancy formation can be prevented, as well as by the structures of the tin-only and lead-only perovskites which can vary with A cation composition.^52^ Further investigation of structural trends in tin–lead perovskites with A cations other than methylammonium, and with careful control of tin vacancies, may reveal whether 50% tin content is a common threshold for structural changes to occur and may develop greater understanding of the intrinsic influences of metal composition on crystal structure.

## 3 Burstein–Moss Effect

**Figure 3:**
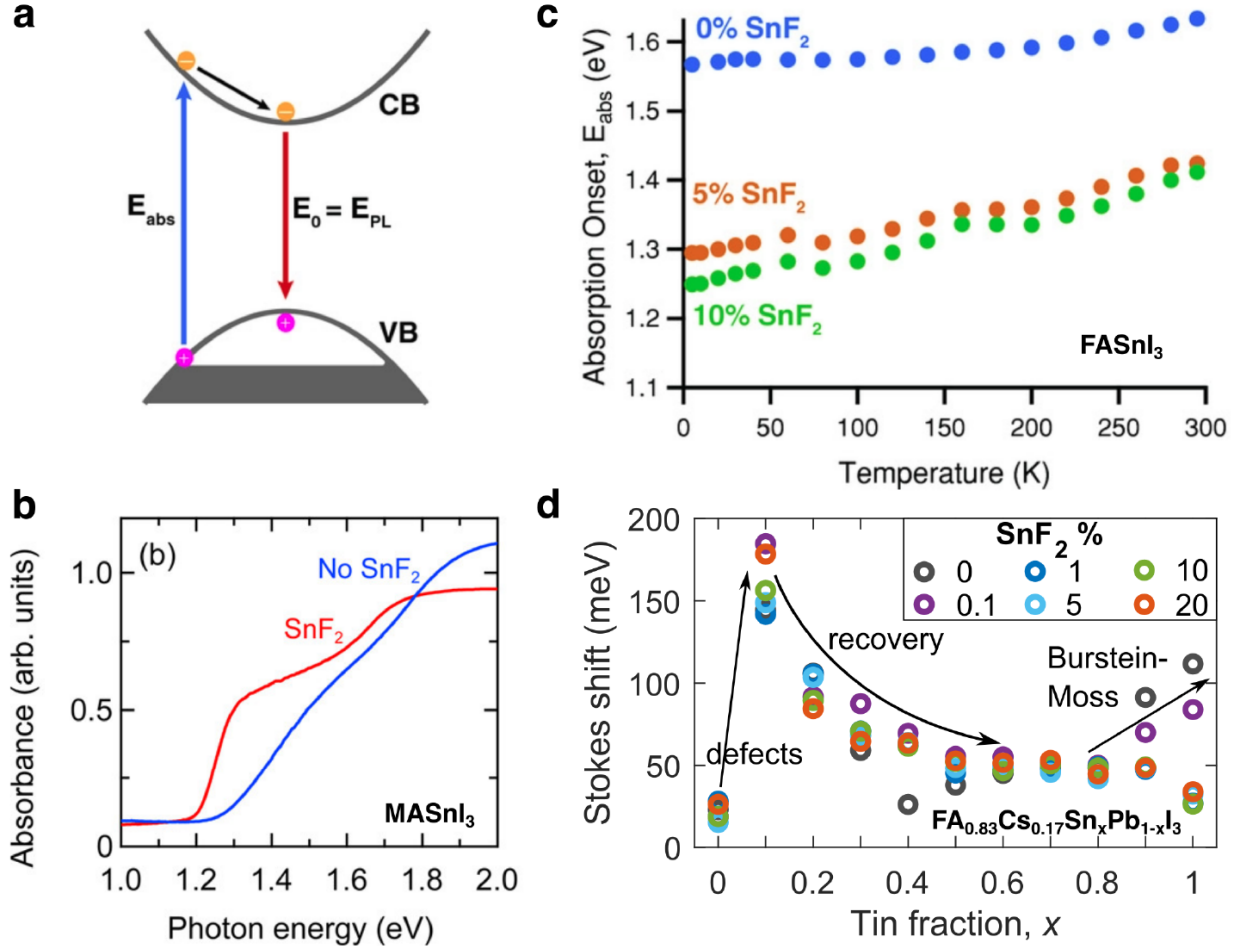
Burstein–Moss effect in tin–lead iodide perovskites with high tin content and large background hole densities owing to tin vacancy defects. (a) Schematic indicating how the resulting lowering of the Fermi level leads to a partial depletion of the valence band and an increase in the energy of the absorption onset *E*_abs_. The PL energy *E*_PL_ is shown to be unaffected because photoexcited electrons will relax to the bottom of the conduction band (CB) and then recombine to fill vacant states at the top of the valence band (VB). [Reprinted with permission from ref (29). Copyright 2018 John Wiley and Sons.] (b) Blue shift in the absorption onset observed for a MASnI_3_ thin film when no SnF_2_ (blue line) had been added during film fabrication, compared to the case when 20% SnF_2_ (red line) had been added to suppress tin vacancy formation. [Reprinted with permission from ref (67). Copyright 2017 American Chemical Society.] (c) Blue-shifted absorption onset energies *E*_abs_ for FASnI_3_ thin films for which 0%, 5%, or 10% SnF_2_ had been added to the precursor with respect to SnI_2_. For 10% SnF_2_, *E*_abs_ approaches the value of the bandgap, and two phase transitions become discernible. [Reprinted with permission from ref (29). Copyright 2018 John Wiley and Sons.] (d) Stokes shift between the absorption onset and emission peak energies for thin films of FA_0.83_Cs_0.17_Sn_*x*_Pb_1–*x*_I_3_ as a function of tin fraction *x* and for a range of SnF_2_ additions. At the high-tin-content end, the Burstein–Moss effect leads to increased Stokes shifts for low SnF_2_ addition, while at the low-tin-content end, small inclusions of tin lead to a defective compositional region. [Adapted with permission from ref (42). Copyright 2020 John Wiley and Sons.]

One peculiarity of tin–lead halide perovskites is their tendency to develop significant blue shifts of the absorption onset in the presence of strong tin vacancy formation.^29,42,67^ Such permanent Burstein–Moss effects^68,69^ arise because significant hole doping causes a downshift of the Fermi-level and a depletion of the electronic states near the top of the valence band (see schematic in Figure 3a). Consequently, electronic transitions between the highest occupied states in the valence band and the conduction band now occur at energies exceeding the intrinsic bandgap energy. Such effects are well known for classical inorganic semiconductors such as InSb, whose absorption onset has been found to blue shift considerably for high doping levels.^68,69^Figure 3b exemplifies how the Burstein–Moss effect modifies the absorption spectra near their onset for MASnI_3_ films produced with and without SnF_2_ additive. Without SnF_2_ present to mitigate tin vacancy formation, the resulting background hole density causes blue-shifted onsets whose oscillator strength is weakened even for photon energies higher up into the band,^67^ possibly as a result of exciton screening. Similar effects are observed for FASnI_3_, for which they are shown to disappear following the addition of as little as 1–5% SnF_2_ during film processing (see Figure 3c,d).^29,42^ Interestingly, the energy of the emission (photoluminescence) peak is hardly affected by the depletion of electronic states near the top of the valence band, because photoexcited electrons first relax to the conduction band edge, then subsequently recombine with holes to fill states near the valence band edge, as indicated schematically in Figure 3a. As a result, the Burstein–Moss effect causes sizable Stokes shifts between absorption and emission in heavily doped tin-rich tin–lead iodide films that may exceed a few hundred meV.^29,42,67^ As Figure 3d illustrates, the most substantive Stokes shifts occur for materials with tin content in excess of 70%, because these are most susceptible to heavy tin vacancy formation.^42^ Interestingly, the Burstein–Moss effect may also interfere with a correct assessment of band bowing from absorption measurements, because the blue-shift in absorption for tin-rich compositions artificially enhances the bowing parameter *b* and lowers the value of tin fraction at which the minimum bandgap is perceived to occur.^42^ Such issues may thus partly contribute to the observed variation in bowing parameter across literature studies, evident in Figure 2.

Overall, the susceptibility of tin-rich perovskites to the Burstein–Moss effect is clearly detrimental for photovoltaic operation, because it will directly translate into open-circuit voltage losses (from the perspective of Stokes shifts) or photocurrent losses (if viewed as an absorption bleach). In addition, any gradual shifts occurring through tin vacancy formation in the energy of the perovskite’s valence band maximum over time may also detrimentally affect its alignment with the energy levels of the hole extractor layer. Therefore, such shifts ultimately present a hurdle to the long-term stability of tin–lead perovskite solar cells, unless they can be reliably prevented from occurring (e.g., by additives such as SnF_2_, or impermeable encapsulation) over the projected lifetime of the device.

## 4 Charge-Carrier Recombination

The prevalence of tin oxidation and background hole doping in tin halide perovskites usually dominates their charge-carrier recombination dynamics. Additional charge-carrier recombination pathways introduced by these extrinsic impurities include both non-radiative Shockley–Read–Hall recombination^21,70^ and pseudo-monomolecular recombination of photoexcited electrons with background holes.^5,20,29,53,71^ The latter process has been shown to be radiative,^71^ being fundamentally identical to that of band-to-band recombination of photogenerated electrons and holes, but scales linearly with the photogenerated charge-carrier density because the density of background holes typically exceeds that of photogenerated holes in these materials.^29,71^ The introduction of such radiative recombination pathways means that tin halide perovskites often display relatively high photoluminescence quantum efficiencies, despite their short charge-carrier lifetimes.^72,73^ Such luminescence enhancement with respect to lead-only counterparts is particularly prominent at low photo-generated charge-carrier densities, given the pseudo-monomolecular nature of the doping-induced radiative recombination.^71^ While the high luminescence efficiency of tin halide perovskites is somewhat of a red herring for photovoltaic devices, it may however be advantageous for light-emitting and lasing applications.^72^

**Figure 4:**
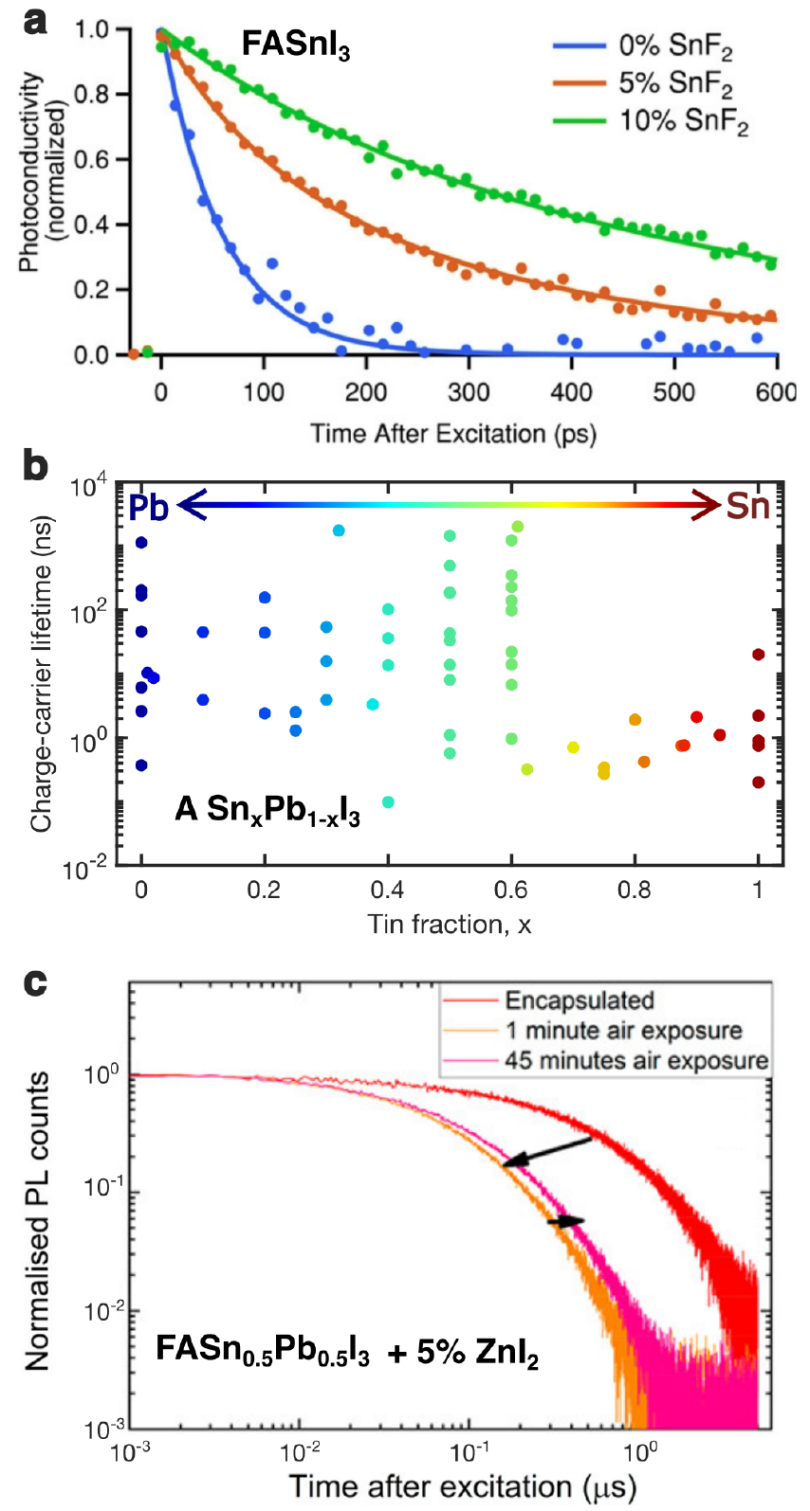
Effect of tin oxidation and vacancy formation on the lifetimes of charge carriers in tin–lead halide perovskites. (a) THz photoconductivity transients for FASnI_3_ thin films with 0%, 5%, or 10% SnF_2_ added during processing, resulting in background hole densities of 2.2 × 10^20^ cm^–3^, 2.0 × 10^19^ cm^–3^, and 7.2 × 10^18^ cm^–3^, respectively. [Adapted with permission from ref (29). Copyright 2018 John Wiley and Sons.] (b) Recombination lifetimes of charge carriers in a range of different tin–lead halide perovskites ASn_*x*_Pb_1–*x*_X_3_, where A is formamidinium, methylammonium, Cs, or a mixture thereof, X is either iodide, bromide, or a mixture thereof. A total of 62 values were extracted from a range of literature studies^5,20,21,32,42−46,53,70,74−78,78−83^ and are shown as a function of tin fraction *x*. In the majority of these studies, unwanted tin vacancy formation was suppressed by the use of SnF_2_, alone^5,42,43,45,46,53,70,75,76,82,83^ or with other additives.^5,20,21,32,44,79^ Long charge-carrier lifetimes are common for lead-only (*x* = 0) perovskites and for tin fraction *x* between ∼30 and 60%. (c) Photoluminescence transients of FASn_0.5_Pb_0.5_I_3_ (5% ZnI_2_ added w.r.t. FAI in precursor) for a film fabricated and encapsulated under inert nitrogen atmosphere (red curve), and 1 min (orange) and 45 min (pink) after the encapsulation had been broken, exposing the film to air. [Adapted from ref (44). American Chemical Society 2019].

The additional charge-carrier recombination pathways introduced by tin oxidation and vacancy formation may to a certain extent be ameliorated through variations in processing, such as the addition of SnF_2_^29,42,74^ (see, e.g., Figure 4a). To examine charge-carrier lifetimes thus achieved for tin–lead halide perovskites, we display in Figure 4b the result of our (non-exhaustive) survey of literature studies (refs (5, 20, 21, 32, 42−46, 53, 70, 74−83)) that yielded 62 values for charge-carrier lifetimes recorded from pulsed-photoexcitation experiments. The majority of these values are for perovskite materials prepared with SnF_2_,^5,42,43,45,46,53,70,75,76,82,83^ sometimes combined with other additives,^5,20,21,32,44,79^ to suppress unwanted tin vacancy formation. Only two of the plotted lifetimes correspond to thin-film samples prepared without such additives,^74,81^ the other exceptions being based on use of a metallic tin precursor,^78^ or tuned crystal growth methods.^77,80^ Interestingly, the figure demonstrates that short charge-carrier lifetimes are prevalent for all tin–lead halide perovskites with tin content in excess of 60%, for which they rarely exceed a few nanoseconds. These findings suggest that current mitigation approaches still fail to fully address tin vacancy formation at the tin-rich end of the compositional range that is particularly prone to such effects. We note that for the popular mitigation technique of adding SnF_2_ to precursor solutions, a cause may be that, while a tin-rich environment reduces the likelihood of tin vacancy formation and hole doping, it may unfortunately also create tin interstitials and iodide vacancies that constitute deep-level traps.^35^ Therefore, neither a tin-poor nor a tin-rich environment will suffice,^42^ and it is unlikely that this approach can ever fully suppress fast recombination pathways in tin-rich tin–lead halide perovskites. However, some promising alternative approaches based on metal substitution have recently been implemented^44^ and examined^35^ which may ultimately succeed in stabilizing these materials. In addition, for intermediate tin–lead compositions, such as the case of FAPb_0.5_Sn_0.5_I_3_ highlighted in Figure 4c, avoiding oxygen exposure at all times through device encapsulation may well suffice to suppress tin vacancy formation.^44^

Interestingly, lead-rich perovskites at the other end of the tin–lead compositional spectrum also exhibit deficiencies in optoelectronic properties, albeit unrelated to tin vacancy formation. Tin–lead iodide perovskites with tin content between 0.5 and 20% display short PL lifetimes, broadened spectra, increased Stokes shifts, a drop in PL quantum yield, and large Urbach tails, compared with their lead-only counterparts.^42,53,82,84^ Such effects can also be seen in our literature survey of mixed tin–lead halide perovskites (Figure 4b) which indicates that for tin content between 0.5 and 20%, charge-carrier lifetimes rarely exceed 100 ns. Addition of minute fractions of tin to lead halide perovskites therefore appears to introduce non-radiative traps that are unlikely to be linked with changing band structure properties, as these vary only gradually from lead to tin halide perovskites.^35^ Instead, such underperformance could potentially be related to the large mismatch in metal-iodide bond lengths deriving from the large ionic size discrepancy of tin compared with lead.^85^ Such mismatch may cause increased energetic disorder,^53,82^ or enhanced polaronic effects^85^ when only small fractions of the much smaller tin are introduced into lead-only iodide perovskites.

Overall, the interplay between these two effects means that mixed tin–lead halide perovskites currently exhibit the highest charge-carrier lifetimes and performance within the relatively narrow compositional range with tin content between 30 and 60%.^82^ As Figure 4b shows, within this range, charge-carrier lifetimes in excess of 1 μs have been achieved on several occasions.^5,20,77^ Fortunately, as Figure 2 illustrates, these compositions also offer the lowest achievable bandgaps for tin–lead halide perovskites, making them highly suitable for bottom cells in multi-junction photovoltaic devices, or high-efficiency single-junction cells.^17−19^ However, the use of tin-only halide perovskites as entirely lead-free absorber layers may well require radical new approaches to boost charge-carrier lifetimes above the currently reported maximum values of at most a few tens of nanoseconds. Progress here will require carefully balanced, stable control of tin content, given that a tin-poor environment causes tin vacancies and ensuing hole doping, while a tin-rich environment generates tin interstitials and iodide vacancies that constitute deep-level traps.^23,35^ Any new approaches on film processing or additives will therefore need to eliminate such trade-offs between formation of these three most prominent defects in tin halide perovskites.

## 5 Charge-Carrier Mobilities

A sufficiently high charge-carrier mobility is a prerequisite for efficient charge-carrier extraction in photovoltaic devices. Intriguingly, tin-rich metal halide perovskites offer the prospect of fundamental charge-carrier mobilities that are significantly higher than those of their lead halide counterparts. Unfortunately, such intrinsic advantages are all too often counteracted by the extrinsic lowering of mobilities in the presence of tin vacancy formation and the associated hole doping. We discuss below the intrinsic and extrinsic mechanisms that combine to govern the mobility of charge carriers in tin–lead halide perovskites.

**Figure 5:**
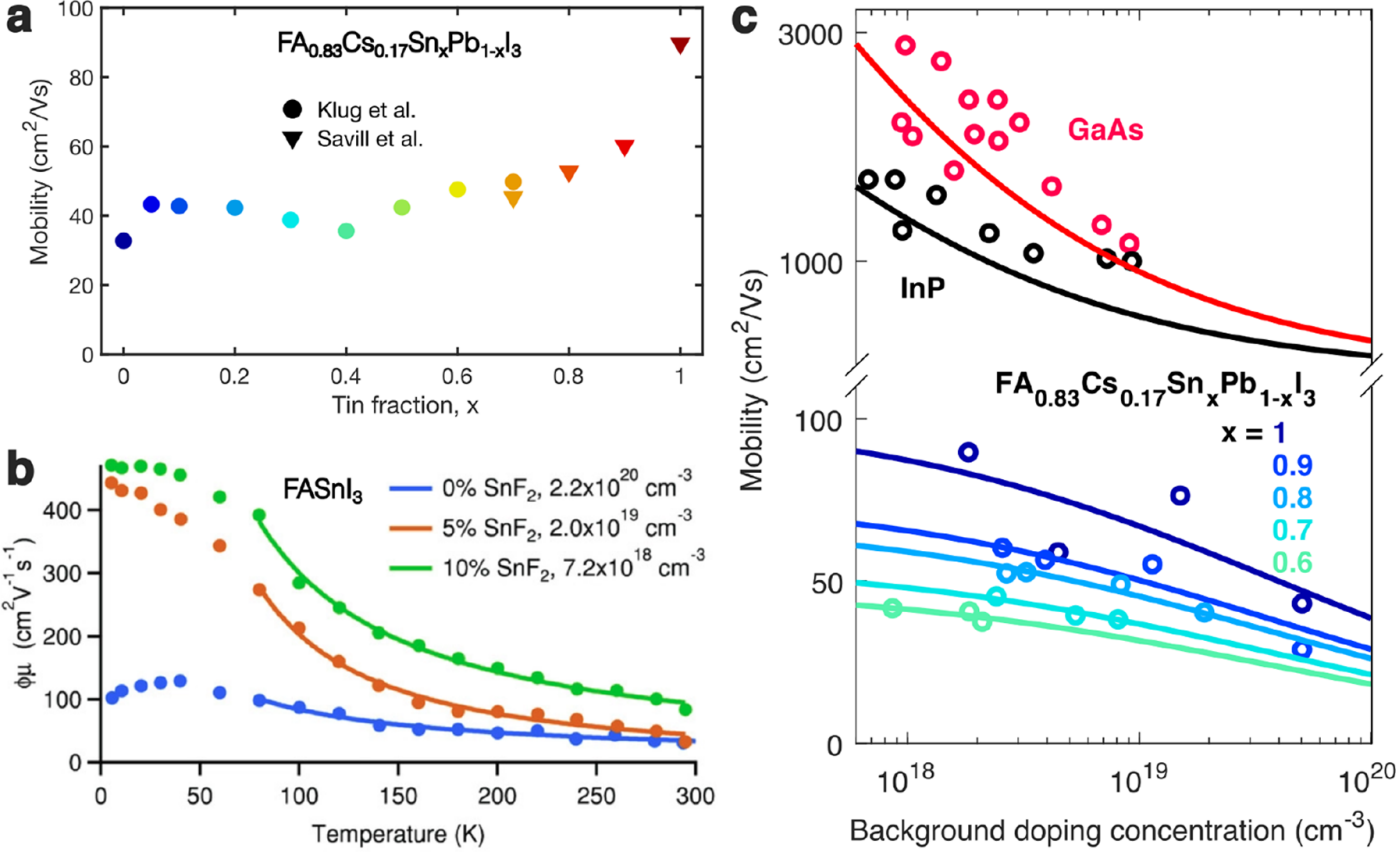
Influence of composition and defects on the charge-carrier mobility in tin–lead iodide perovskites. (a) Change in THz electron–hole sum mobility with tin fraction *x* in FA_0.83_Cs_0.17_Sn_*x*_Pb_1–*x*_I_3_ thin films fabricated with 10% SnF_2_ added to the precursor solution to suppress tin vacancy formation. Data were extracted from two separate sets of films, reported in ref (82) (filled circle markers) and ref (42) (filled triangle markers). (b) Temperature-dependent THz electron–hole sum mobility for FASnI_3_ thin films with 0%, 5%, or 10% SnF_2_ added during processing, resulting in background hole densities as stated in the legend. Solid lines represent fits according to a *T*^m^ dependence, yielding *m* = −0.8, −1.4, and −1.1, respectively. [Reprinted with permission from ref (29). Copyright 2018 John Wiley and Sons.] (c) Change in THz electron–hole sum mobility as a function of background hole density induced by unintentional doping (tin vacancies) in FA_0.83_Cs_0.17_Sn_*x*_Pb_1–*x*_I_3_ films with various tin fractions *x*,^42^ compared to that reported previously for two inorganic semiconductors, GaAs and InP,^98^ and fitted with the empirical formula by Hilsum.^98^ [Reprinted with permission from ref (42). John Wiley and Sons 2020]

The fundamental limit to the charge-carrier mobilities of tin–lead halide perovskites derives from interactions between charge carriers and longitudinal optical (LO) phonons of the polar metal halide lattice,^86,87^ captured in Fröhlich’s theory.^88−90^ Experimental evidence for the dominance of this mechanism comes from analysis of the temperature dependence of spectral emission broadening^67,86^ and charge-carrier mobilities.^29,91−93^ As the example in Figure 5b shows, the charge-carrier mobility in FASnI_3_ rises with decreasing temperature, suggesting that for sufficiently high-quality films, the charge-carrier mobility is governed by coupling to phonon modes. Within the Fröhlich model, charge-carrier motion is impeded because the macroscopic electric field generated by a longitudinal optical phonon interacts with charge carriers, leading to a local lattice distortion around the charge, termed a “large polaron”.^88−90^ The resulting mobility μ of a charge carrier is inversely proportional to the coupling constant α = ϵ_Fr_^–1^(Ry/*ℏ*ω_LO_)^1/2^(m*/*m*_e_)^1/2^, where ω_LO_ is the LO phonon energy, Ry = 13.606 eV the Rydberg constant, m*/*m*_e_ the effective mass m* of the charge carrier as a fraction of the free electron mass *m*_e_, and ϵ_Fr_^–1^ = ϵ_∞_^–1^ – ϵ_static_^–1^ is determined by the static and high-frequency limits of the dielectric function with respect to the LO phonon resonance.^89,90,94,95^ Therefore, expected trends across a series of tin–lead iodide perovskites can be readily estimated from changes in values of the dielectric function, LO phonon energies, and effective masses. From such considerations, a significant enhancement in charge-carrier mobilities should be expected toward the tin-rich end of the series.^95^ Effective masses of charge carriers drop appreciably^36,95^ when moving from lead- to tin-based perovskites, commensurate with the lowering of the bandgap. In addition, optical phonon modes upshift in frequency toward the tin-rich compounds, as recently observed experimentally^42^ and expected theoretically^36,95^ for the lighter atomic mass of tin compared with lead. Since the Fröhlich mobility increases with the dimensionless parameter β = *ℏ*ω_LO_/*k*_B_*T*, an upshift in LO phonon frequencies will effectively shift temperature-dependent mobility curves to higher temperatures, thus the room-temperature mobility is enhanced. Such trends of increasing mobilities with increasing tin content can indeed be observed for carefully passivated tin–lead perovskite films made as part of a single fabrication and measurement series, as illustrated in Figure 5a. They may also be discerned in careful literature surveys,^87^ which have highlighted higher cross-study averages of charge-carrier mobilities in tin iodide perovskites compared with lead iodide perovskites.

Despite such discernible underlying trends, charge-carrier mobility values reported for tin iodide perovskites vary by 3 orders of magnitude between different studies,^26−28,30,31,38,42,51,96,97^ highlighting a significant influence of extrinsic effects linked to fabrication techniques (as well as variations in measurement protocols^87^). For such tin-rich compositions, a particularly strong influence again derives from tin vacancy formation, which, as discussed above, causes background hole densities as high as 10^17^–10^20^ cm^–3^. The remnant dopant site (the tin vacancy) must be negatively charged to preserve charge neutrality, and consequently acts as a scattering site to charge carriers, lowering their mobilities. Figures 1a and 5b demonstrate how addition of SnF_2_ during the fabrication process causes a substantial increase in charge-carrier mobilities by lowering the materials’ propensity toward tin vacancy formation and the resulting scattering.^28,29^ Such effects are also evident in the temperature dependence of the charge-carrier mobility for thin films of FASnI_3_, illustrated in Figure 5b. The presence of ionized tin defects leads to shallower rises in mobility toward low temperature^29^ because Coulombic interactions with such impurities become more effective as the thermal velocity of charge carriers is slowed.^88,98^ Such lowering of charge-carrier mobility with increasing doping concentration is also well known to occur for a range of inorganic semiconductors.^98^Figure 5c contrasts the changes in charge-carrier mobility observed^42^ across the tin-rich end of a FA_0.83_Cs_0.17_Sn_*x*_Pb_1–*x*_I_3_ series with those recorded previously for GaAs and InP.^98^ As indicated by the solid lines, all of these semiconductors can be well-described by the Hilsum formula^98^ which assumes the mobility of charge carriers to be limited by coupling to phonons and scattering off ionized impurities.

Overall, while charge-carrier mobilities are fundamentally enhanced with increasing tin content in tin–lead iodide perovskites, the concomitant susceptibility to tin vacancy formation and the resulting scattering of carriers with these ionized impurities may instead lower the mobilities for highly defective materials. To enhance and preserve charge-carrier extraction efficiencies in photovoltaic cells, it is thus essential for these materials to be stabilized against tin vacancy formation.

## 6 Exciton Binding Energies

The exciton binding energy *E*_b_ plays a crucial role for a semiconductor’s suitability as a light absorber in photovoltaic applications. On the one hand, values of *E*_b_ below thermal energies (26 meV at room temperature) are desirable because bound electron–hole pairs (excitons) may then self-dissociate, allowing efficient photocurrent collection of electrons and holes to their respective extraction layers. On the other hand, a low binding energy weakens the absorption coefficient strength near the band edge, because Elliott theory^99^ dictates that Coulomb correlations also enhance the oscillator strength of above-gap continuum states that are populated by free charge carriers. As a result, materials with low *E*_b_ have more gradual absorption onsets, requiring thicker layers that lower charge-carrier extraction efficiencies. On balance, exciton binding energies falling somewhat but not too far below thermal energies are therefore ideal for photovoltaic applications.

In metal halide perovskites, excitons are generally well-described by the hydrogenic model of the “Wannier” exciton, whose binding energy is given by^94,99^
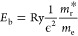
2where Ry = 13.606 eV is the Rydberg constant, ϵ is the value of the dielectric function, and *m*_r_^*^/*m*_e_ is the reduced effective mass of the electron–hole system, expressed as a fraction of the free electron mass *m*_e_. Unsurprisingly, the exciton binding energy for the prototypical lead iodide perovskite MAPbI_3_ has already been intensely investigated,^100−107^ with a recent survey^94^ compiling a room-temperature literature average of 12 ± 7 meV. However, experimental investigations of exciton binding energies in tin halide and tin–lead halide perovskites are still relatively scarce,^29,108,109^ mostly because of issues with sample stability and self-doping discussed above.

Given these experimental difficulties, we begin by discussing theoretical expectations of changes in exciton binding energy when tin is substituted for lead within a series of tin–lead halide perovskites. As eq 2 shows, *E*_b_ depends on the reduced effective mass *m*_r_^*^ and the value of ϵ, which may vary along the tin–lead perovskite series. First-principles calculations have suggested that the reduced effective mass of charge carriers should drop appreciably (by 20–50%) when moving from lead-iodide perovskites to their tin-iodide counterparts,^36,95^ as typical for a lower bandgap material.^88^ Magneto-absorption measurements conducted at 2 K have confirmed such trends experimentally, finding a fall of *m*_r_^*^ by about 25% as tin content increases from *x* = 0.2 to 0.8 in MAPb_1–*x*_Sn_*x*_I_3_ films.^108^ From effective-mass considerations alone, the exciton binding energy for tin iodide perovskite would therefore be expected to be lower than for lead-iodide perovskites.

An evaluation of the second critical parameter, ϵ, is considerably more complicated because ϵ is a particularly strong function of frequency in metal halide perovskites,^94^ opening a debate^101,110,111^ on which value of ϵ should enter eq 2. Self-consistency is an important criterion here,^94,101,112^ which requires that when a directly determined value of the exciton binding energy is used to derive a value of ϵ according to eq 2, that value of ϵ must then indeed be encountered at the frequency *E*_b_/*h*. In this context, it is also important to assess whether the energies of optical phonons fall above or below the value of *E*_b_, since in the latter case, phonons may no longer be able to follow the motion of the electron–hole pair effectively^113^ leading to lower screening and therefore lower effective values of ϵ entering eq 2. Optical phonon modes for mixed tin–lead iodide perovskites have recently been shown to increase in frequency with increasing tin content, as would be expected for lighter tin atoms.^42^ As a result, excitons in tin iodide perovskites would thus experience more effective screening by the ionic tin halide lattice, leading again to lower exciton binding energies than those encountered in lead-based counterparts.^112,114^ First-principles calculations by Umari et al. based on such self-consistent approaches have indeed suggested that ϵ should increase appreciably with increasing tin content along the tin–lead iodide perovskite series, with the exciton binding energy expected to reduce by over a factor of 2 from lead to tin iodide perovskites.^112^

**Figure 6:**
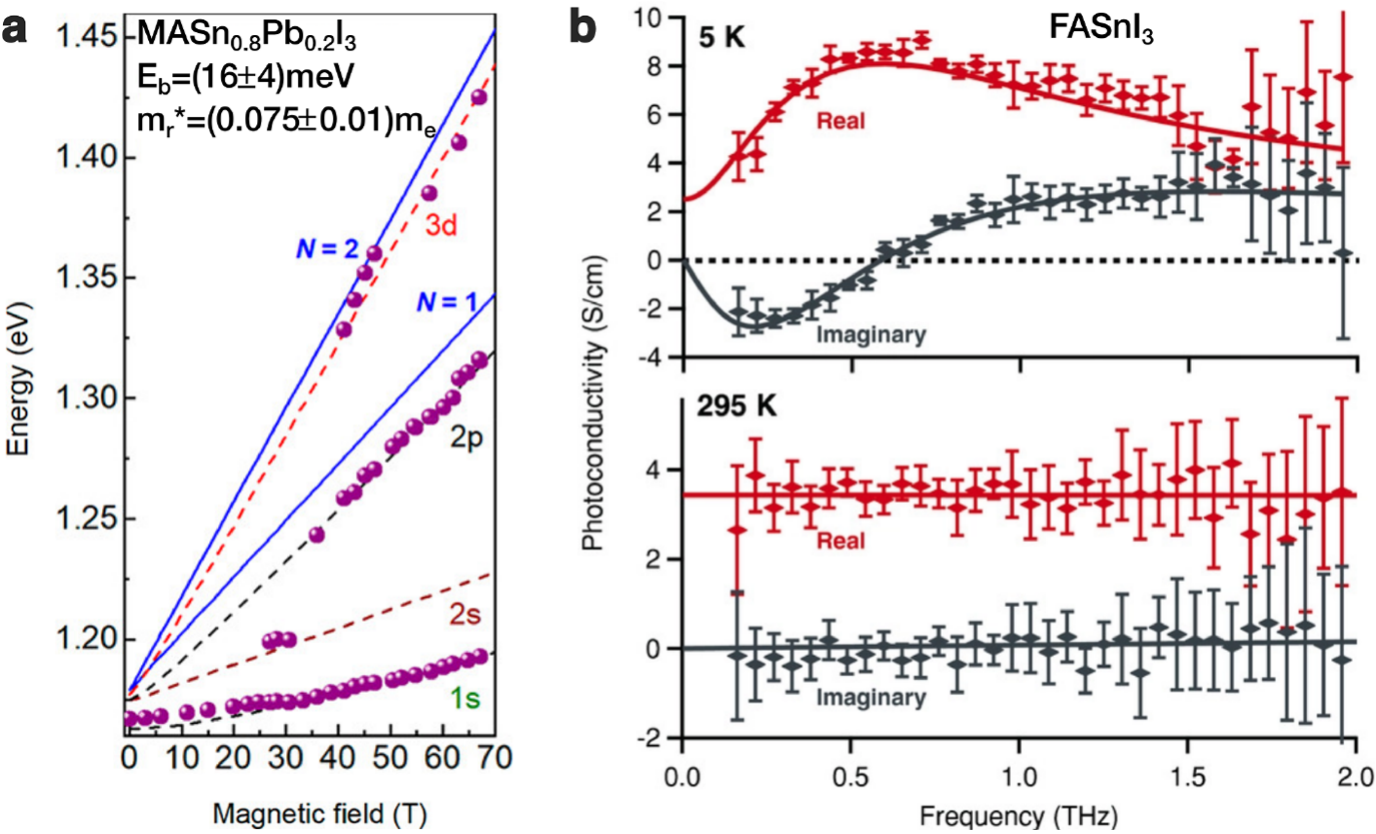
Exciton binding energy determination in tin–lead iodide perovskites. (a) Landau energy level fan chart extracted from magneto-absorption measurements on a MASn_0.8_Pb_0.2_I_3_ film at a temperature of 2 K. Solid lines show fits from which an effective reduced mass of around 0.075 free-electron masses and an exciton binding energy (Rydberg value) of 16 meV (viewed to be an upper limit) were extracted. [Adapted from ref (108). Copyright 2019 American Chemical Society.] (b) Photoinduced complex THz conductivity spectra for a FASnI_3_ (10% SnF_2_) film at temperatures of 5 K (top) and 295 K (bottom). At low temperatures, the spectra are dominated by 1s-to-2p intra-excitonic transitions, yielding an exciton binding energy of 3.1 meV. At room temperature, excitons are dissociated, yielding only a Drude-like free-carrier response. [Reprinted with permission from ref (29). Copyright 2018 John Wiley and Sons.]

Direct experimental probes of exciton binding energies in tin–lead halide perovskites are still relatively scarce because of complications arising from spontaneous self-doping and the ensuing disorder in these materials. Galkowski et al. conducted low-temperature magneto-absorption measurements at high magnetic fields to examine how *E*_b_ varies with tin content *x* in MAPb_1–*x*_Sn_*x*_I_3_ films.^108^ Unfortunately, the authors found that the intrinsic inhomogeneity and instability of these materials meant that magneto-absorption features were less well resolved than in their earlier^101^ work on lead halide perovskites. Therefore, discernible higher-lying excitonic features were only visible at very high magnetic field (≥40 T) which made the required extrapolation to zero fields through fan charts unreliable (see Figure 6a). The authors determined a constant value of *E*_b_ = 16 meV for tin fractions *x* = 0.2, 0.6, and 0.8, similar to the value they had determined earlier for lead-only counterparts. Such invariance of *E*_b_ with tin content would be surprising, given the theoretical considerations outlined above. However, as a result of the measurement uncertainties, the authors suggest that these values only comprise upper limits of the actual exciton binding energies in tin–lead perovskites.^101^ Another direct approach to determining an exciton binding energy was made by Milot et al.^29^ for FASnI_3_ thin films through examining photoinduced changes in the THz spectral range at low temperature (5 K). They observed a Lorentzian oscillator resonance feature in the complex transmission spectra that they ascribed to inter-excitonic transitions, given that these features only appeared at low temperature, were independent of excitation fluence, and disappeared in heavily doped films in which excitons would be screened (see Figure 6b). By evaluation of the resonance energy, an exciton binding energy of only 3.1 meV was extracted. Finally, attempts have been made^109^ to extract exciton binding energies from fits of Elliott’s theory to the absorption onset of evaporated thin films of FA_0.75_Cs_0.25_Pb_0.45_Sn_0.55_I_3_. While such fits also yielded low values of *E*_*b*_ of the order of several meV, the extracted values varied between samples and fraction of SnF_2_ added in the deposition process. For tin-rich tin–lead halide perovskites, in particular, the Burstein–Moss effect, electronic screening and energetic disorder deriving from self-doping may complicate determination of exciton binding energies through the usual Elliott method.

To summarize these considerations, exciton binding energies are theoretically expected to fall as tin content is increased along the tin–lead halide perovskite series, because of a lowering of charge-carrier masses, and an increase in optical phonon frequencies that facilitates effective screening of Coulomb interactions by the polar sublattice. The resulting positive correlation between exciton binding energy and bandgap energy is well known from the case of classic inorganic semiconductors.^88^ Further direct measurements of the exciton binding energies in tin-rich tin–lead iodide perovskites would be helpful, given that present studies in the field still appear to be inconclusive, with some pointing toward lower exciton binding energies for tin-rich perovskites,^29,109^ while others suggest that the binding energy may potentially be unchanged along the tin–lead iodide perovskite series.^108^ Finally, we note that both lead and tin iodide perovskites appear to exhibit exciton binding energies sufficiently below thermal energies at room temperatures to induce efficient charge-carrier separation.

## 7 Charge-Carrier Cooling

For tin halide perovskites, the cooling dynamics of charge carriers following above-gap excitation and thermalization have been an interesting subject of debate, following early reports^115^ of unusually long (nanoseconds) cooling times in FASnI_3_. Prolonged cooling dynamics could facilitate the long-term goal of hot-carrier solar cells, in which charge carriers are extracted before the excess energy supplied during above-gap photoexcitation has been transferred to the lattice, permitting PCEs above the Shockley–Queisser limit.^116^ The time scale on which hot carriers return to the ambient lattice temperature has been observed to vary widely across different metal halide perovskite compositions.^117^ The unusually slow cooling^115^ initially proposed for tin iodide perovskites prompted suggestions that these could be exceptional candidate materials for realizing hot carrier extraction in solar cells.^118^ However, a subsequent investigation instead attributed the nanosecond dynamics observed at the high-energy end of FASnI_3_ to slow relaxation between energetically disordered states connected to the oxidation of tin, an extrinsic effect.^119^ It is therefore still debated whether these nanosecond dynamics truly reflect a population of hot carriers that could be extracted to increase device voltage.

Meanwhile, when such dynamics are examined in tin–lead halide perovskites over the picosecond time scales during which charge-carrier cooling has typically been found to occur in lead halide perovskites^105,117^ and inorganic semiconductors, such as GaAs,^120^ the small number of studies conducted so far have reached somewhat inconclusive results. While Savill et al.^119^ and Verma et al.^121^ reported slowed cooling for tin halide perovskites compared with their lead-based counterparts, Monti et al.^97^ reported the opposite, and Ma et al.^74^ similar time scales. Verma et al. found slowed picosecond cooling rates for increasing tin content along a mixed tin–lead perovskite series and attributed these effects to slower phonon emission deriving from two effects, stronger screening as tin addition increases the dielectric constant and deformation potential scattering arising from changes in lattice distortion and band structure.^121^ However, Monti et al. observed the opposite trend in initial cooling dynamics despite the use of similar excitation conditions, finding shorter cooling times with increasing tin concentration up to 75%. They stipulated that cooling in this Fröhlich regime was dominated by longitudinal optical phonon emission as the dominant mechanism, and attributed the observed acceleration of cooling to an increasing frequency of phonon modes as the lighter tin cation was introduced.^97^

Overall, it is clear that further investigation will be required to provide a complete understanding of how composition influences the time scales of charge-carrier cooling in tin–lead halide perovskites. Experimental studies have yielded highly disparate results to date,^74,97,115,119,121^ most likely because of differences in both sample quality and the methods by which charge-carrier temperatures are extracted from measured data. Since the propensity of tin-rich perovskites toward tin vacancy formation creates large background charge-carrier densities, additional inter-carrier scattering pathways may become available that will accelerate the loss rate of excess energy. Here it is worth noting that such large densities of background holes comprise already thermalized “cold” charge carriers which thus offer rapid thermalization pathways to newly photogenerated (“hot”) charge carriers. In addition, the energetic disorder caused by the presence of ionized tin vacancies may cause energetic relaxation of charge-carriers slowly migrating through high-energy tails of the available density of states. Such relaxation dynamics may mimic charge-carrier cooling dynamics when examined at the high-energy end of emission peaks, despite being unrelated to the actual cooling processes.^119^ Disentangling such disorder-related effects from true charge-carrier cooling dynamics is thus important for a correct experimental determination of cooling time scales. Finally, theoretical investigations may further help to elucidate charge-carrier cooling in these materials, drawing, e.g., on the knowledge available for trends of charge-carrier mobilities across the tin–lead perovskite series, which are also governed by electron–phonon interactions.

## Summary and Future Directions

Our analysis shows that, from a fundamental perspective, mixed tin–lead halide perovskites have much to offer compared with their lead-only counterparts. Toward the tin-rich end of the compositional range, fundamentally attainable mobilities rise, exciton binding energies are expected to fall, and charge-carrier cooling may potentially slow, all of which are potentially beneficial for photovoltaic applications. In addition, significant bandgap bowing allows for attractive near-infrared bandgaps at intermediate tin content, ideally suited for applications in high-efficiency single-junction photovoltaic cells, or in bottom cells for multi-junction devices. Several challenges still remain with regard to our understanding of the underlying optoelectronic properties of these materials. A full literature consensus on experimentally determined exciton binding energies and hot-carrier cooling dynamics has not yet been reached. A theoretical evaluation of exciton binding energies fully from first-principles approaches is still a complex task because it requires evaluation of the difference between electronic transitions in the presence and absence of Coulomb correlations, which is difficult to achieve accurately when such differences are comparatively small. First-principles calculations specifically for mixed perovskite stoichiometries are computationally demanding, because of the need for large supercells to accurately reflect different compositions and the resulting configurational disorder. Nevertheless, the general picture emerging at this point is that the intrinsic optoelectronic properties of tin–lead halide perovskites are highly suited to efficient solar cell operation.

In reality, the most challenging aspect, therefore, remains attaining control over the extrinsic defect chemistry of tin–lead halide perovskites. As our review has highlighted, the propensity of tin-rich compositions toward tin oxidation, vacancy formation, and the ensuing unintentional background doping with holes has many adverse effects on their real-world optoelectronic performance. At high tin vacancy density, the Burstein–Moss effect leads to blue shifts of absorption onsets that lower light-harvesting efficiencies and potentially cause energetic misalignment with extraction layers, with adverse effects on the open-circuit voltages and photocurrents of solar cells. Tin vacancy formation further accelerates charge-carrier recombination significantly, by causing enhanced non-radiative Shockley–Read–Hall recombination and radiative pseudo-monomolecular recombination of photogenerated electrons with a large pool of background holes. Moreover, when tin vacancies release holes into the valence band, they become negatively charged, acting as local scattering sites and lowering the mobility of charge carriers. Therefore, the combined lowering of charge-carrier lifetimes and mobilities results in significantly reduced charge-carrier diffusion lengths in tin–lead halide perovskites exhibiting high tin vacancy densities.

Nevertheless, impressive performance of solar cells has been achieved when mixed tin–lead halide perovskites of intermediate tin content (30–60%) have been incorporated into photovoltaic devices.^17−21^ Our review shows that in this intermediate range, tin–lead perovskite materials offer the best of both worlds: lowest attainable bandgaps as a result of bandgap bowing, charge-carrier lifetimes similar to those of lead-only counterparts (often in excess of microseconds) and moderate charge-carrier mobility enhancements over those of lead halide perovskites. However, it remains to be seen whether such enhanced performance will indeed be stable over decades, in particular given the instability of the Sn^2+^ oxidative state in the presence of oxygen. In addition, as the prevalence of tin vacancy mediated defects recedes with better material processing and passivation protocols emerging, other defect-mediated recombination pathways may come to our attention. In this context, it is interesting to note that inclusion of only a low fraction of tin into lead halide perovskite leads to highly defective materials,^82^ for reasons that are not yet fully understood. In addition, it is becoming apparent that the currently most popular technique to prevent tin vacancy formation, i.e., SnF_2_ addition, may unfortunately introduce additional non-radiative recombination pathways^29^ through local tin-rich environments that cause tin interstitials and iodide vacancies, which constitute deep-level traps.^35^ Since this approach may therefore fail to fully suppress fast charge-carrier recombination pathways, new passivation strategies are urgently required, which may for example include metal substitution.^35,44^

Finally, fully lead-free tin iodide perovskites (ASnI_3_) still suffer from tin vacancy formation that is relatively ill-controlled. Our literature survey discussed above indicates that charge-carrier lifetimes rarely exceed a few nanoseconds, and perhaps unsurprisingly, best single-cell PCEs have so far been much lower than those for lead-only counterparts, with recently reported values reaching slightly above 13%.^83,122^ While such PCEs are far below the theoretically attainable single-junction limit,^10,11^ it is worth acknowledging that better-performing lead-free contenders have yet to emerge from the plethora of metal halide semiconductors explored to date.^13^ Attaining long-term, sustainable control over tin vacancy formation in lead-free tin iodide perovskites, and its detrimental effects on bandgaps, charge-carrier lifetimes, and mobilities, is therefore perhaps the most challenging goal of all.

## References

[ref1] Roy P.; Sinha N. K.; Tiwari S.; Khare A. A Review on Perovskite Solar Cells: Evolution of Architecture, Fabrication Techniques, Commercialization Issues and Status. Sol. Energy 2020, 198, 665–688. 10.1016/j.solener.2020.01.080.

[ref2] De Wolf S.; Holovsky J.; Moon S.-J.; Löper P.; Niesen B.; Ledinsky M.; Haug F.-J.; Yum J.-H.; Ballif C. Organometallic Halide Perovskites: Sharp Optical Absorption Edge and its Relation to Photovoltaic Performance. J. Phys. Chem. Lett. 2014, 5, 1035–1039. 10.1021/jz500279b. 26270984

[ref3] Stranks S. D.; Eperon G. E.; Grancini G.; Menelaou C.; Alcocer M. J. P.; Leijtens T.; Herz L. M.; Petrozza A.; Snaith H. J. Electron-Hole Diffusion Lengths Exceeding 1 Micrometer in an Organometal Trihalide Perovskite Absorber. Science 2013, 342, 341–344. 10.1126/science.1243982. 24136964

[ref4] Wehrenfennig C.; Eperon G. E.; Johnston M. B.; Snaith H. J.; Herz L. M. High Charge Carrier Mobilities and Lifetimes in Organolead Trihalide Perovskites. Adv. Mater. 2014, 26, 1584–1589. 10.1002/adma.201305172. 24757716 PMC4722848

[ref5] Yang Z.; Yu Z.; Wei H.; Xiao X.; Ni Z.; Chen B.; Deng Y.; Habisreutinger S. N.; Chen X.; Wang K.; Zhao J.; Rudd P. N.; Berry J. J.; Beard M. C.; Huang J. Enhancing Electron Diffusion Length in Narrow-Bandgap Perovskites for Efficient Monolithic Perovskite Tandem Solar Cells. Nat. Commun. 2019, 10, 4498 10.1038/s41467-019-12513-x. 31582749 PMC6776504

[ref6] Xing G.; Mathews N.; Lim S. S.; Yantara N.; Liu X.; Sabba D.; Grätzel M.; Mhaisalkar S.; Sum T. C. Low-Temperature Solution-Processed Wavelength-Tunable Perovskites for Lasing. Nat. Mater. 2014, 13, 476–480. 10.1038/nmat3911. 24633346

[ref7] Kim J.; Lee S.-H.; Lee J. H.; Hong K.-H. The Role of Intrinsic Defects in Methylammonium Lead Iodide Perovskite. J. Phys. Chem. Lett. 2014, 5, 1312–1317. 10.1021/jz500370k. 26269973

[ref8] Ball J. M.; Petrozza A. Defects in Perovskite-Halides and their Effects in Solar Cells. Nat. Energy 2016, 1, 16149 10.1038/nenergy.2016.149.

[ref9] Eperon G. E.; Stranks S. D.; Menelaou C.; Johnston M. B.; Herz L. M.; Snaith H. J. Formamidinium Lead Trihalide: a Broadly Tunable Perovskite for Efficient Planar Heterojunction Solar Cells. Energy Environ. Sci. 2014, 7, 982 10.1039/c3ee43822h.

[ref10] Ruppel W.; Würfel P. Upper Limit for the Conversion of Solar Energy. IEEE Trans. Electron Devices 1980, 27, 877–882. 10.1109/T-ED.1980.19950.

[ref11] Shockley W.; Queisser H. J. Detailed Balance Limit of Efficiency of *p* – *n* Junction Solar Cells. J. Appl. Phys. 1961, 32, 510–519. 10.1063/1.1736034.

[ref12] Babayigit A.; Ethirajan A.; Muller M.; Conings B. Toxicity of Organometal Halide Perovskite Solar Cells. Nat. Mater. 2016, 15, 247–251. 10.1038/nmat4572. 26906955

[ref13] Ke W.; Kanatzidis M. G. Prospects for Low-Toxicity Lead-Free Perovskite Solar Cells. Nat. Commun. 2019, 10, 965 10.1038/s41467-019-08918-3. 30814499 PMC6393492

[ref14] Unger E. L.; Kegelmann L.; Suchan K.; Sörell D.; Korte L.; Albrecht S. Roadmap and Roadblocks for the Band Gap Tunability of Metal Halide Perovskites. J. Mater. Chem. A 2017, 5, 11401–11409. 10.1039/C7TA00404D.

[ref15] Filip M. R.; Giustino F. The Geometric Blueprint of Perovskites. Proc. Natl. Acad. Sci. U. S. A. 2018, 115, 5397–5402. 10.1073/pnas.1719179115. 29735683 PMC6003477

[ref16] Xiao Z.; Song Z.; Yan Y. From Lead Halide Perovskites to Lead-Free Metal Halide Perovskites and Perovskite Derivatives. Adv. Mater. 2019, 31, 1803792 10.1002/adma.201803792. 30680809

[ref17] Wang C.; Song Z.; Li C.; Zhao D.; Yan Y. Low-Bandgap Mixed Tin-Lead Perovskites and Their Applications in All-Perovskite Tandem Solar Cells. Adv. Funct. Mater. 2019, 29, 1808801 10.1002/adfm.201808801.

[ref18] Gu S.; Lin R.; Han Q.; Gao Y.; Tan H.; Zhu J. Tin and Mixed Lead–Tin Halide Perovskite Solar Cells: Progress and their Application in Tandem Solar Cells. Adv. Mater. 2020, 32, 1907392 10.1002/adma.201907392. 32053273

[ref19] Leijtens T.; Bush K. A.; Prasanna R.; McGehee M. D. Opportunities and Challenges for Tandem Solar Cells using Metal Halide Perovskite Semiconductors. Nat. Energy 2018, 3, 828–838. 10.1038/s41560-018-0190-4.

[ref20] Tong J.; et al. Carrier Lifetimes of > 1 μs in Sn-Pb Perovskites Enable Efficient All-Perovskite Tandem Solar Cells. Science 2019, 364, 475–479. 10.1126/science.aav7911. 31000592

[ref21] Xiao K.; et al. All-Perovskite Tandem Solar cells with 24.2% Certified Efficiency and Area over 1 cm^2^ using Surface-Anchoring Zwitterionic Antioxidant. Nat. Energy 2020, 5, 870–880. 10.1038/s41560-020-00705-5.

[ref22] Zhou X.; Zhang L.; Wang X.; Liu C.; Chen S.; Zhang M.; Li X.; Yi W.; Xu B. Highly Efficient and Stable GABr-Modified Ideal-Bandgap (1.35 eV) Sn/Pb Perovskite Solar Cells Achieve 20.63% Efficiency with a Record Small V_*OC*_ Deficit of 0.33 V. Adv. Mater. 2020, 32, 1908107 10.1002/adma.201908107. 32100401

[ref23] Xu P.; Chen S.; Xiang H.-J.; Gong X.-G.; Wei S.-H. Influence of Defects and Synthesis Conditions on the Photovoltaic Performance of Perovskite Semiconductor CsSnI_3_. Chem. Mater. 2014, 26, 6068–6072. 10.1021/cm503122j.

[ref24] Leijtens T.; Prasanna R.; Gold-Parker A.; Toney M. F.; McGehee M. D. Mechanism of Tin Oxidation and Stabilization by Lead Substitution in Tin Halide Perovskites. ACS Energy Lett. 2017, 2, 2159–2165. 10.1021/acsenergylett.7b00636.

[ref25] Lanzetta L.; Aristidou N.; Haque S. A. Stability of Lead and Tin Halide Perovskites: The Link between Defects and Degradation. J. Phys. Chem. Lett. 2020, 11, 574–585. 10.1021/acs.jpclett.9b02191. 31913050

[ref26] Mitzi D. B.; Feild C. A.; Schlesinger Z.; Laibowitz R. B. Transport, Optical, and Magnetic Properties of the Conducting Halide Perovskite CH_3_NH_3_SnI_3_. J. Solid State Chem. 1995, 114, 159–163. 10.1006/jssc.1995.1023.

[ref27] Takahashi Y.; Hasegawa H.; Takahashi Y.; Inabe T. Hall Mobility in Tin Iodide Perovskite CH_3_NH_3_SnI_3_: Evidence for a Doped Semiconductor. J. Solid State Chem. 2013, 205, 39–43. 10.1016/j.jssc.2013.07.008.

[ref28] Kumar M. H.; Dharani S.; Leong W. L.; Boix P. P.; Prabhakar R. R.; Baikie T.; Shi C.; Ding H.; Ramesh R.; Asta M.; Graetzel M.; Mhaisalkar S. G.; Mathews N. Lead-Free Halide Perovskite Solar Cells with High Photocurrents Realized Through Vacancy Modulation. Adv. Mater. 2014, 26, 7122–7127. 10.1002/adma.201401991. 25212785

[ref29] Milot R. L.; Klug M. T.; Davies C. L.; Wang Z.; Kraus H.; Snaith H. J.; Johnston M. B.; Herz L. M. The Effects of Doping Density and Temperature on the Optoelectronic Properties of Formamidinium Tin Triiodide Thin Films. Adv. Mater. 2018, 30, 1804506 10.1002/adma.201804506. 30222220

[ref30] Stoumpos C. C.; Malliakas C. D.; Kanatzidis M. G. Semiconducting Tin and Lead Iodide Perovskites with Organic Cations: Phase Transitions, High Mobilities, and Near-Infrared Photoluminescent Properties. Inorg. Chem. 2013, 52, 9019–9038. 10.1021/ic401215x. 23834108

[ref31] Noel N. K.; Stranks S. D.; Abate A.; Wehrenfennig C.; Guarnera S.; Haghighirad A.-A.; Sadhanala A.; Eperon G. E.; Pathak S. K.; Johnston M. B.; Petrozza A.; Herz L. M.; Snaith H. J. Lead-Free Organic–Inorganic Tin Halide Perovskites for Photovoltaic Applications. Energy Environ. Sci. 2014, 7, 3061–3068. 10.1039/C4EE01076K.

[ref32] Lin R.; Xiao K.; Qin Z.; Han Q.; Zhang C.; Wei M.; Saidaminov M. I.; Gao Y.; Xu J.; Xiao M.; Li A.; Zhu J.; Sargent E. H.; Tan H. Monolithic All-Perovskite Tandem Solar Cells with 24.8% Efficiency Exploiting Comproportionation to Suppress Sn(II) Oxidation in Precursor Ink. Nat. Energy 2019, 4, 864–873. 10.1038/s41560-019-0466-3.

[ref33] Meng X.; Wu T.; Liu X.; He X.; Noda T.; Wang Y.; Segawa H.; Han L. Highly Reproducible and Efficient FASnI_3_ Perovskite Solar Cells Fabricated with Volatilizable Reducing Solvent. J. Phys. Chem. Lett. 2020, 11, 2965–2971. 10.1021/acs.jpclett.0c00923. 32216309

[ref34] Dalpian G. M.; Liu Q.; Stoumpos C. C.; Douvalis A. P.; Balasubramanian M.; Kanatzidis M. G.; Zunger A. Changes in Charge Density vs Changes in Formal Oxidation States: The Case of Sn Halide Perovskites and their Ordered Vacancy Analogues. Phys. Rev. Mater. 2017, 1, 025401 10.1103/PhysRevMaterials.1.025401.

[ref35] Meggiolaro D.; Ricciarelli D.; Alasmari A. A.; Alasmary F. A. S.; De Angelis F. Tin versus Lead Redox Chemistry Modulates Charge Trapping and Self-Doping in Tin/Lead Iodide Perovskites. J. Phys. Chem. Lett. 2020, 11, 3546–3556. 10.1021/acs.jpclett.0c00725. 32298590

[ref36] Umari P.; Mosconi E.; De Angelis F. Relativistic GW Calculations on CH_3_NH_3_PbI_3_ and CH_3_NH_3_SnI_3_ Perovskites for Solar Cell Applications. Sci. Rep. 2015, 4, 4467 10.1038/srep04467. PMC5394751 24667758

[ref37] Even J.; Pedesseau L.; Jancu J.; Katan C. DFT and *k•p* Modelling of the Phase Transitions of Lead and Tin Halide Perovskites for Photovoltaic Cells. Phys. Status Solidi RRL 2014, 8, 31–35. 10.1002/pssr.201308183.

[ref38] Chung I.; Song J.-H.; Im J.; Androulakis J.; Malliakas C. D.; Li H.; Freeman A. J.; Kenney J. T.; Kanatzidis M. G. CsSnI_3_: Semiconductor or Metal? High Electrical Conductivity and Strong Near-Infrared Photoluminescence from a Single Material. High Hole Mobility and Phase-Transitions. J. Am. Chem. Soc. 2012, 134, 8579–8587. 10.1021/ja301539s. 22578072

[ref39] Qiu X.; Cao B.; Yuan S.; Chen X.; Qiu Z.; Jiang Y.; Ye Q.; Wang H.; Zeng H.; Liu J.; Kanatzidis M. G. From Unstable CsSnI_3_ to Air-Stable Cs_2_SnI_6_: A Lead-Free Perovskite Solar Cell Light Absorber with Bandgap of 1.48 eV and High Absorption Coefficient. Sol. Energy Mater. Sol. Cells 2017, 159, 227–234. 10.1016/j.solmat.2016.09.022.

[ref40] Xiao M.; Gu S.; Zhu P.; Tang M.; Zhu W.; Lin R.; Chen C.; Xu W.; Yu T.; Zhu J. Tin-Based Perovskite with Improved Coverage and Crystallinity through Tin-Fluoride-Assisted Heterogeneous Nucleation. Adv. Opt. Mater. 2018, 6, 1700615 10.1002/adom.201700615.

[ref41] Liao W.; Zhao D.; Yu Y.; Grice C. R.; Wang C.; Cimaroli A. J.; Schulz P.; Meng W.; Zhu K.; Xiong R.-G.; Yan Y. Lead-Free Inverted Planar Formamidinium Tin Triiodide Perovskite Solar Cells Achieving Power Conversion Efficiencies up to 6.22%. Adv. Mater. 2016, 28, 9333–9340. 10.1002/adma.201602992. 27571446

[ref42] Savill K. J.; Ulatowski A. M.; Farrar M. D.; Johnston M. B.; Snaith H. J.; Herz L. M. Impact of Tin Fluoride Additive on the Properties of Mixed Tin-Lead Iodide Perovskite Semiconductors. Adv. Funct. Mater. 2020, 30, 2005594 10.1002/adfm.202005594.

[ref43] Yuan J.; Jiang Y.; He T.; Shi G.; Fan Z.; Yuan M. Two-Dimensional Perovskite Capping Layer for Stable and Efficient Tin-Lead Perovskite Solar Cells. Sci. China: Chem. 2019, 62, 629–636. 10.1007/s11426-018-9436-1.

[ref44] Bowman A. R.; Klug M. T.; Doherty T. A. S.; Farrar M. D.; Senanayak S. P.; Wenger B.; Divitini G.; Booker E. P.; Andaji-Garmaroudi Z.; Macpherson S.; Ruggeri E.; Sirringhaus H.; Snaith H. J.; Stranks S. D. Microsecond Carrier Lifetimes, Controlled p-Doping, and Enhanced Air Stability in Low-Bandgap Metal Halide Perovskites. ACS Energy Lett. 2019, 4, 2301–2307. 10.1021/acsenergylett.9b01446. 31544151 PMC6748266

[ref45] Xu X.; Chueh C.-C.; Yang Z.; Rajagopal A.; Xu J.; Jo S. B.; Jen A. K.-Y. Ascorbic Acid as an Effective Antioxidant Additive to Enhance the Efficiency and Stability of Pb/Sn-Based Binary Perovskite Solar Cells. Nano Energy 2017, 34, 392–398. 10.1016/j.nanoen.2017.02.040.

[ref46] Jayawardena K. D. G. I.; et al. Approaching the Shockley-Queisser Limit for Fill Factors in Lead-Tin Mixed Perovskite Photovoltaics. J. Mater. Chem. A 2020, 8, 693–705. 10.1039/C9TA10543C.

[ref47] Johnston M. B.; Herz L. M. Hybrid Perovskites for Photovoltaics: Charge-Carrier Recombination, Diffusion and Radiative Efficiencies. Acc. Chem. Res. 2016, 49, 146–154. 10.1021/acs.accounts.5b00411. 26653572

[ref48] Diau E. W. G.; Jokar E.; Rameez M. Strategies to Improve Performance and Stability for Tin-Based Perovskite Solar Cells. ACS Energy Lett. 2019, 4, 1930–1937. 10.1021/acsenergylett.9b01179.

[ref49] Yao H.; Zhou F.; Li Z.; Ci Z.; Ding L.; Jin Z. Strategies for Improving the Stability of Tin-Based Perovskite (ASnX_3_) Solar Cells. Adv. Sci. 2020, 7, 1903540 10.1002/advs.201903540. PMC7237862 32440480

[ref50] Li B.; Chang B.; Pan L.; Li Z.; Fu L.; He Z.; Yin L. Tin-Based Defects and Passivation Strategies in Tin-Related Perovskite Solar Cells. ACS Energy Lett. 2020, 5, 3752–3772. 10.1021/acsenergylett.0c01796.

[ref51] Eperon G. E.; et al. Perovskite-Perovskite Tandem Photovoltaics with Optimized Band Gaps. Science 2016, 354, 861–865. 10.1126/science.aaf9717. 27856902

[ref52] Prasanna R.; Gold-Parker A.; Leijtens T.; Conings B.; Babayigit A.; Boyen H.-G.; Toney M. F.; McGehee M. D. Band Gap Tuning Via Lattice Contraction and Octahedral Tilting in Perovskite Materials for Photovoltaics. J. Am. Chem. Soc. 2017, 139, 11117–11124. 10.1021/jacs.7b04981. 28704048

[ref53] Parrott E. S.; Green T.; Milot R. L.; Johnston M. B.; Snaith H. J.; Herz L. M. Interplay of Structural and Optoelectronic Properties in Formamidinium Mixed Tin–Lead Triiodide Perovskites. Adv. Funct. Mater. 2018, 28, 1802803 10.1002/adfm.201802803.

[ref54] Hao F.; Stoumpos C. C.; Chang R. P. H.; Kanatzidis M. G. Anomalous Band Gap Behavior in Mixed Sn and Pb Perovskites Enables Broadening of Absorption Spectrum in Solar Cells. J. Am. Chem. Soc. 2014, 136, 8094–8099. 10.1021/ja5033259. 24823301

[ref55] Zhao B.; Abdi-Jalebi M.; Tabachnyk M.; Glass H.; Kamboj V. S.; Nie W.; Pearson A. J.; Puttisong Y.; Gödel K. C.; Beere H. E.; Ritchie D. A.; Mohite A. D.; Dutton S. E.; Friend R. H.; Sadhanala A. High Open-Circuit Voltages in Tin-Rich Low-Bandgap Perovskite-Based Planar Heterojunction Photovoltaics. Adv. Mater. 2017, 29, 1604744 10.1002/adma.201604744. 28066989

[ref56] Liu C.; Fan J.; Li H.; Zhang C.; Mai Y. Highly Efficient Perovskite Solar Cells with Substantial Reduction of Lead Content. Sci. Rep. 2016, 6, 35705 10.1038/srep35705. 27752138 PMC5067674

[ref57] Rajagopal A.; Stoddard R. J.; Hillhouse H.; Jen A. K. Y. On Understanding Bandgap Bowing and Optoelectronic Quality in Pb-Sn Alloy Hybrid Perovskites. J. Mater. Chem. A 2019, 7, 16285–16293. 10.1039/C9TA05308E.

[ref58] Vurgaftman I.; Meyer J. R.; Ram-Mohan L. R. Band Parameters for III–V Compound Semiconductors and their Alloys. J. Appl. Phys. 2001, 89, 5815–5875. 10.1063/1.1368156.

[ref59] Schwartz H. A.; Laurenzen H.; Marzouk A.; Runkel M.; Brinkmann K. O.; Rogalla D.; Riedl T.; Ashhab S.; Olthof S. Band-Gap Tuning in All-Inorganic CsPb_*x*_Sn_1–*x*_Br_3_ Perovskites. ACS Appl. Mater. Interfaces 2021, 13, 4203–4210. 10.1021/acsami.0c20285. 33435668

[ref60] Noh J. H.; Im S. H.; Heo J. H.; Mandal T. N.; Seok S. I. Chemical Management for Colorful, Efficient, and Stable Inorganic–Organic Hybrid Nanostructured Solar Cells. Nano Lett. 2013, 13, 1764–1769. 10.1021/nl400349b. 23517331

[ref61] Im J.; Stoumpos C. C.; Jin H.; Freeman A. J.; Kanatzidis M. G. Antagonism between Spin–Orbit Coupling and Steric Effects Causes Anomalous Band Gap Evolution in the Perovskite Photovoltaic Materials CH_3_NH_3_Sn_1–*x*_Pb_*x*_I_3_. J. Phys. Chem. Lett. 2015, 6, 3503–3509. 10.1021/acs.jpclett.5b01738. 27120685

[ref62] Khatun S.; Maiti A.; Pal A. J. Bowing of Transport Gap in Hybrid Halide Perovskite Alloys (CH_3_NH_3_Sn_1–*x*_Pb_*x*_I_3_): Which Band is Responsible?. Appl. Phys. Lett. 2020, 116, 012104 10.1063/1.5134749.

[ref63] Goyal A.; McKechnie S.; Pashov D.; Tumas W.; Van Schilfgaarde M.; Stevanović V. Origin of Pronounced Nonlinear Band Gap Behavior in Lead–Tin Hybrid Perovskite Alloys. Chem. Mater. 2018, 30, 3920–3928. 10.1021/acs.chemmater.8b01695.

[ref64] Valadares F.; Guilhon I.; Teles L. K.; Marques M. Atomistic Origins of Enhanced Band Gap, Miscibility, and Oxidation Resistance in α-CsPb_1–*x*_Sn_*x*_I_3_ Mixed Perovskite. J. Phys. Chem. C 2020, 124, 26124–26133. 10.1021/acs.jpcc.0c07356.

[ref65] Anaya M.; Correa-Baena J. P.; Lozano G.; Saliba M.; Anguita P.; Roose B.; Abate A.; Steiner U.; Gratzel M.; Calvo M. E.; Hagfeldt A.; Miguez H. Optical Analysis of CH_3_NH_3_Sn_*x*_Pb_1–*x*_I_3_ Absorbers: a Roadmap for Perovskite-on-Perovskite Tandem Solar Cells. J. Mater. Chem. A 2016, 4, 11214–11221. 10.1039/C6TA04840D. PMC5059782 27774148

[ref66] Zong Y.; Wang N.; Zhang L.; Ju M.-G.; Zeng X. C.; Sun X. W.; Zhou Y.; Padture N. P. Homogenous Alloys of Formamidinium Lead Triiodide and Cesium Tin Triiodide for Efficient Ideal-Bandgap Perovskite Solar Cells. Angew. Chem., Int. Ed. 2017, 56, 12658–12662. 10.1002/anie.201705965. 28671739

[ref67] Handa T.; Yamada T.; Kubota H.; Ise S.; Miyamoto Y.; Kanemitsu Y. Photocarrier Recombination and Injection Dynamics in Long-Term Stable Lead-Free CH_3_NH_3_SnI_3_ Perovskite Thin Films and Solar Cells. J. Phys. Chem. C 2017, 121, 16158–16165. 10.1021/acs.jpcc.7b06199.

[ref68] Burstein E. Anomalous Optical Absorption Limit in InSb. Phys. Rev. 1954, 93, 632–633. 10.1103/PhysRev.93.632.

[ref69] Moss T. S. The Interpretation of the Properties of Indium Antimonide. Proc. Phys. Soc., London, Sect. B 1954, 67, 775–782. 10.1088/0370-1301/67/10/306.

[ref70] Li C.; Song Z.; Zhao D.; Xiao C.; Subedi B.; Shrestha N.; Junda M. M.; Wang C.; Jiang C.-S.; Al-Jassim M.; Ellingson R. J.; Podraza N. J.; Zhu K.; Yan Y.; et al. Reducing Saturation-Current Density to Realize High-Efficiency Low-Bandgap Mixed Tin–Lead Halide Perovskite Solar Cells. Adv. Energy Mater. 2019, 9, 1803135 10.1002/aenm.201803135.

[ref71] Milot R. L.; Eperon G. E.; Green T.; Snaith H. J.; Johnston M. B.; Herz L. M. Radiative Monomolecular Recombination Boosts Amplified Spontaneous Emission in HC(NH_2_)_2_SnI_3_ Perovskite Films. J. Phys. Chem. Lett. 2016, 7, 4178–4184. 10.1021/acs.jpclett.6b02030. 27715054

[ref72] Xing G.; Kumar M. H.; Chong W. K.; Liu X.; Cai Y.; Ding H.; Asta M.; Grätzel M.; Mhaisalkar S.; Mathews N.; Sum T. C. Solution–Processed Tin–Based Perovskite for Near-Infrared Lasing. Adv. Mater. 2016, 28, 8191–8196. 10.1002/adma.201601418. 27417520

[ref73] Poli I.; Kim G.-W.; Wong E. L.; Treglia A.; Folpini G.; Petrozza A. High External Photoluminescence Quantum Yield in Tin Halide Perovskite Thin Films. ACS Energy Lett. 2021, 6, 609–611. 10.1021/acsenergylett.0c02612. 33614965 PMC7887870

[ref74] Ma L.; Hao F.; Stoumpos C. C.; Phelan B. T.; Wasielewski M. R.; Kanatzidis M. G. Carrier Diffusion Lengths of over 500 nm in Lead-Free Perovskite CH_3_NH_3_SnI_3_ Films. J. Am. Chem. Soc. 2016, 138, 14750–14755. 10.1021/jacs.6b09257. 27750426

[ref75] Liao W.; Zhao D.; Yu Y.; Shrestha N.; Ghimire K.; Grice C. R.; Wang C.; Xiao Y.; Cimaroli A. J.; Ellingson R. J.; Podraza N. J.; Zhu K.; Xiong R. G.; Yan Y. Fabrication of Efficient Low-Bandgap Perovskite Solar Cells by Combining Formamidinium Tin Iodide with Methylammonium Lead Iodide. J. Am. Chem. Soc. 2016, 138, 12360–12363. 10.1021/jacs.6b08337. 27622903

[ref76] Yang Z.; Zhang X.; Yang W.; Eperon G. E.; Ginger D. S. Tin-Lead Alloying for Efficient and Stable All-Inorganic Perovskite Solar Cells. Chem. Mater. 2020, 32, 2782–2794. 10.1021/acs.chemmater.9b04265.

[ref77] Ju D.; Dang Y.; Zhu Z.; Liu H.; Chueh C.-C.; Li X.; Wang L.; Hu X.; Jen A. K.-Y.; Tao X. Tunable Band Gap and Long Carrier Recombination Lifetime of Stable Mixed CH_3_NH_3_Pb_*x*_Sn_1–*x*_Br_3_ Single Crystals. Chem. Mater. 2018, 30, 1556–1565. 10.1021/acs.chemmater.7b04565.

[ref78] Zhu Z.; Li N.; Zhao D.; Wang L.; Jen A. K.-Y. Improved Efficiency and Stability of Pb/Sn Binary Perovskite Solar Cells Fabricated by Galvanic Displacement Reaction. Adv. Energy Mater. 2019, 9, 1802774 10.1002/aenm.201802774.

[ref79] Ripolles T. S.; Yamasuso D.; Zhang Y.; Kamarudin M. A.; Ding C.; Hirotani D.; Shen Q.; Hayase S. New Tin(II) Fluoride Derivative as a Precursor for Enhancing the Efficiency of Inverted Planar Tin/Lead Perovskite Solar Cells. J. Phys. Chem. C 2018, 122, 27284–27291. 10.1021/acs.jpcc.8b09609.

[ref80] Selvarajan P.; Kundu K.; Sathish C. I.; Umapathy S.; Vinu A. Enriched Photophysical Properties and Thermal Stability of Tin(II) Substituted Lead-Based Perovskite Nanocrystals with Mixed Organic–Inorganic Cations. J. Phys. Chem. C 2020, 124, 9611–9621. 10.1021/acs.jpcc.0c02223.

[ref81] Hu M.; Chen M.; Guo P.; Zhou H.; Deng J.; Yao Y.; Jiang Y.; Gong J.; Dai Z.; Zhou Y.; Qian F.; Chong X.; Feng J.; Schaller R. D.; Zhu K.; Padture N. P.; Zhou Y.; et al. Sub-1.4 eV Bandgap Inorganic Perovskite Solar Cells with Long-Term Stability. Nat. Commun. 2020, 11, 151 10.1038/s41467-019-13908-6. 31919343 PMC6952449

[ref82] Klug M. T.; et al. Metal Composition Influences Optoelectronic Quality in Mixed-Metal Lead–Tin Triiodide Perovskite Solar Absorbers. Energy Environ. Sci. 2020, 13, 1776–1787. 10.1039/D0EE00132E.

[ref83] Nishimura K.; Kamarudin M. A.; Hirotani D.; Hamada K.; Shen Q.; Iikubo S.; Minemoto T.; Yoshino K.; Hayase S. Lead-free Tin-halide Perovskite Solar Cells with 13% Efficiency. Nano Energy 2020, 74, 104858 10.1016/j.nanoen.2020.104858.

[ref84] Subedi B.; Li C.; Junda M. M.; Song Z.; Yan Y.; Podraza N. J. Effects of Intrinsic and Atmospherically Induced Defects in Narrow Bandgap (FASnI_3_)_*x*_(MAPbI_3_)_1–*x*_ Perovskite Films and Solar Cells. J. Chem. Phys. 2020, 152, 064705 10.1063/1.5126867. 32061228

[ref85] Mahata A.; Meggiolaro D.; De Angelis F. From Large to Small Polarons in Lead, Tin, and Mixed Lead–Tin Halide Perovskites. J. Phys. Chem. Lett. 2019, 10, 1790–1798. 10.1021/acs.jpclett.9b00422. 30922057

[ref86] Wright A. D.; Verdi C.; Milot R. L.; Eperon G. E.; Pérez-Osorio M. A.; Snaith H. J.; Giustino F.; Johnston M. B.; Herz L. M. Electron-Phonon Coupling in Hybrid Lead Halide Perovskites. Nat. Commun. 2016, 7, 11755 10.1038/ncomms11755. PMC4894981 27225329

[ref87] Herz L. M. Charge-Carrier Mobilities in Metal Halide Perovskites: Fundamental Mechanisms and Limits. ACS Energy Lett. 2017, 2, 1539–1548. 10.1021/acsenergylett.7b00276.

[ref88] Yu P. Y.; Cardona M. Fundamentals of Semiconductors, 1st ed.; Springer, 1996.

[ref89] Fröhlich H. Electrons in Lattice Fields. Adv. Phys. 1954, 3, 325–361. 10.1080/00018735400101213.

[ref90] Feynman R. P. Slow Electrons in a Polar Crystal. Phys. Rev. 1955, 97, 660–665. 10.1103/PhysRev.97.660.

[ref91] Milot R. L.; Eperon G. E.; Snaith H. J.; Johnston M. B.; Herz L. M. Temperature-Dependent Charge-Carrier Dynamics in CH_3_NH_3_PbI_3_. Adv. Funct. Mater. 2015, 25, 6218–6227. 10.1002/adfm.201502340.

[ref92] Oga H.; Saeki A.; Ogomi Y.; Hayase S.; Seki S. Improved Understanding of the Electronic and Energetic Landscapes of Perovskite Solar Cells: High Local Charge Carrier Mobility, Reduced Recombination, and Extremely Shallow Traps. J. Am. Chem. Soc. 2014, 136, 13818–13825. 10.1021/ja506936f. 25188538

[ref93] Savenije T. J.; Ponseca C. S.; Kunneman L.; Abdellah M.; Zheng K.; Tian Y.; Zhu Q.; Canton S. E.; Scheblykin I. G.; Pullerits T.; Yartsev A.; Sundström V. Thermally Activated Exciton Dissociation and Recombination Control the Carrier Dynamics in Organometal Halide Perovskite. J. Phys. Chem. Lett. 2014, 5, 2189–2194. 10.1021/jz500858a. 26279532

[ref94] Herz L. M. How Lattice Dynamics Moderate the Electronic Properties of Metal-Halide Perovskites. J. Phys. Chem. Lett. 2018, 9, 6853–6863. 10.1021/acs.jpclett.8b02811. 30422667

[ref95] Ponce S.; Schlipf M.; Giustino F. Origin of Low Carrier Mobilities in Halide Perovskites. ACS Energy Lett. 2019, 4, 456–463. 10.1021/acsenergylett.8b02346.

[ref96] Badrooj M.; Jamali-Sheini F.; Torabi N. Optoelectronic Properties of Mixed Sn/Pb Perovskite Solar Cells: The Study of Compressive Strain by Raman Modes. J. Phys. Chem. C 2020, 124, 27136–27147. 10.1021/acs.jpcc.0c07999.

[ref97] Monti M.; Jayawardena K. D. G. I.; Butler-Caddle E.; Bandara R. M. I.; Woolley J. M.; Staniforth M.; Silva S. R. P.; Lloyd-Hughes J. Hot Carriers in Mixed Pb-Sn Halide Perovskite Semiconductors Cool Slowly While Retaining Their Electrical Mobility. Phys. Rev. B: Condens. Matter Mater. Phys. 2020, 102, 245204 10.1103/PhysRevB.102.245204.

[ref98] Hilsum C. Simple Empirical Relationship Between Mobility and Carrier Concentration. Electron. Lett. 1974, 10, 259–260. 10.1049/el:19740205.

[ref99] Elliott R. J. Intensity of Optical Absorption by Excitons. Phys. Rev. 1957, 108, 1384–1389. 10.1103/PhysRev.108.1384.

[ref100] Miyata A.; Mitioglu A.; Plochocka P.; Portugall O.; Wang J. T.-W.; Stranks S. D.; Snaith H. J.; Nicholas R. J. Direct Measurement of the Exciton Binding Energy and Effective Masses for Charge Carriers in an Organic-Inorganic Tri-Halide Perovskite. Nat. Phys. 2015, 11, 582–587. 10.1038/nphys3357.

[ref101] Galkowski K.; Mitioglu A.; Miyata A.; Plochocka P.; Portugall O.; Eperon G. E.; Wang J. T.-W.; Stergiopoulos T.; Stranks S. D.; Snaith H. J.; Nicholas R. J. Determination of the Exciton Binding Energy and Effective Masses for Methylammonium and Formamidinium Lead Tri-Halide Perovskite Semiconductors. Energy Environ. Sci. 2016, 9, 962–970. 10.1039/C5EE03435C.

[ref102] Davies C. L.; Filip M. R.; Patel J. B.; Crothers T. W.; Verdi C.; Wright A. D.; Milot R. L.; Giustino F.; Johnston M. B.; Herz L. M. Bimolecular Recombination in Methylammonium Lead Triiodide Perovskite is an Inverse Absorption Process. Nat. Commun. 2018, 9, 293 10.1038/s41467-017-02670-2. 29348550 PMC5773627

[ref103] Yamada Y.; Nakamura T.; Endo M.; Wakamiya A.; Kanemitsu Y. Photoelectronic Responses in Solution-Processed Perovskite CH_3_NH_3_PbI_3_ Solar Cells Studied by Photoluminescence and Photoabsorption Spectroscopy. IEEE J. Photovoltaics 2015, 5, 401–405. 10.1109/JPHOTOV.2014.2364115.

[ref104] Sestu N.; Cadelano M.; Sarritzu V.; Chen F.; Marongiu D.; Piras R.; Mainas M.; Quochi F.; Saba M.; Mura A.; Bongiovanni G. Absorption F-Sum Rule for the Exciton Binding Energy in Methylammonium Lead Halide Perovskites. J. Phys. Chem. Lett. 2015, 6, 4566–4572. 10.1021/acs.jpclett.5b02099. 26517760

[ref105] Yang Y.; Ostrowski D. P.; France R. M.; Zhu K.; van de Lagemaat J.; Luther J. M.; Beard M. C. Observation of a Hot-Phonon Bottleneck in Lead-Iodide Perovskites. Nat. Photonics 2016, 10, 53–59. 10.1038/nphoton.2015.213.

[ref106] Even J.; Pedesseau L.; Katan C. Analysis of Multivalley and Multibandgap Absorption and Enhancement of Free Carriers Related to Exciton Screening in Hybrid Perovskites. J. Phys. Chem. C 2014, 118, 11566–11572. 10.1021/jp503337a.

[ref107] Soufiani A. M.; Huang F.; Reece P.; Sheng R.; Ho-Baillie A.; Green M. A. Polaronic Exciton Binding Energy in Iodide and Bromide Organic-Inorganic Lead Halide Perovskites. Appl. Phys. Lett. 2015, 107, 231902 10.1063/1.4936418.

[ref108] Galkowski K.; Surrente A.; Baranowski M.; Zhao B.; Yang Z.; Sadhanala A.; Mackowski S.; Stranks S. D.; Plochocka P. Excitonic Properties of Low-Band-Gap Lead–Tin Halide Perovskites. ACS Energy Lett. 2019, 4, 615–621. 10.1021/acsenergylett.8b02243.

[ref109] Ball J.; Buizza L.; Sansom H.; Farrar M.; Klug M.; Borchert J.; Patel J. B.; Herz L. M.; Johnston M. B.; Snaith H. J. Dual-source Co-evaporation of Low-bandgap FA_1–*x*_Cs_*x*_Sn_1–*y*_Pb_*y*_I_3_ Perovskites for Photovoltaics. ACS Energy Lett. 2019, 4, 2748–2756. 10.1021/acsenergylett.9b01855.

[ref110] Herz L. M. Charge Carrier Dynamics in Organic-Inorganic Metal Halide Perovskites. Annu. Rev. Phys. Chem. 2016, 67, 65–89. 10.1146/annurev-physchem-040215-112222. 26980309

[ref111] Lin Q.; Armin A.; Nagiri R. C. R.; Burn P. L.; Meredith P. Electro-Optics of Perovskite Solar Cells. Nat. Photonics 2015, 9, 106–112. 10.1038/nphoton.2014.284.

[ref112] Umari P.; Mosconi E.; De Angelis F. Infrared Dielectric Screening Determines the Low Exciton Binding Energy of Metal-Halide Perovskites. J. Phys. Chem. Lett. 2018, 9, 620–627. 10.1021/acs.jpclett.7b03286. 29336156

[ref113] Klingshirn C. F. Semiconductor Optics, 1st ed.; Springer, 1997; pp 165–166.

[ref114] Bokdam M.; Sander T.; Stroppa A.; Picozzi S.; Sarma D. D.; Franchini C.; Kresse G. Role of Polar Phonons in the Photo Excited State of Metal Halide Perovskites. Sci. Rep. 2016, 6, 28618 10.1038/srep28618. 27350083 PMC4923852

[ref115] Fang H.-H.; Adjokatse S.; Shao S.; Even J.; Loi M. A. Long-Lived Hot-Carrier Light Emission and Large Blue Shift in Formamidinium Tin Triiodide Perovskites. Nat. Commun. 2018, 9, 243 10.1038/s41467-017-02684-w. 29339814 PMC5770436

[ref116] Ross R. T.; Nozik A. J. Efficiency of Hot–Carrier Solar Energy Converters. J. Appl. Phys. 1982, 53, 3813 10.1063/1.331124.

[ref117] Li M.; Fu J.; Xu Q.; Sum T. C. Slow Hot-Carrier Cooling in Halide Perovskites: Prospects for Hot-Carrier Solar Cells. Adv. Mater. 2019, 31, 1802486 10.1002/adma.201802486. 30600555

[ref118] Kahmann S.; Loi M. A. Hot Carrier Solar Cells and the Potential of Perovskites for Breaking the Shockley–Queisser Limit. J. Mater. Chem. C 2019, 7, 2471–2486. 10.1039/C8TC04641G.

[ref119] Savill K. J.; Klug M. T.; Milot R. L.; Snaith H. J.; Herz L. M. Charge-Carrier Cooling and Polarization Memory Loss in Formamidinium Tin Triiodide. J. Phys. Chem. Lett. 2019, 10, 6038–6047. 10.1021/acs.jpclett.9b02353. 31545045

[ref120] Leheny R. F.; Shah J.; Fork R. L.; Shank C. V.; Migus A. Dynamics of Hot Carrier Cooling in Photo-excited GaAs. Solid State Commun. 1979, 31, 809–813. 10.1016/0038-1098(79)90393-4.

[ref121] Verma S. D.; Gu Q.; Sadhanala A.; Venugopalan V.; Rao A. Slow Carrier Cooling in Hybrid Pb–Sn Halide Perovskites. ACS Energy Lett. 2019, 4, 736–740. 10.1021/acsenergylett.9b00251.

[ref122] Wang C.; Zhang Y.; Gu F.; Zhao Z.; Li H.; Jiang H.; Bian Z.; Liu Z. Illumination Durability and High-Efficiency Sn-based Perovskite Solar Cell under Coordinated Control of Phenylhydrazine and Halogen Ions. Matter 2021, 4, 709–721. 10.1016/j.matt.2020.11.012.

